# Transdiagnostic Assessment of Temporal Experience (TATE) in Mental Disorders—Empirical Validation and Adaptation of a Structured Phenomenological Interview

**DOI:** 10.3390/jcm13154325

**Published:** 2024-07-24

**Authors:** Anastazja Szuła, Marcin Moskalewicz, Giovanni Stanghellini

**Affiliations:** 1 Philosophy of Mental Health Unit, Department of Social Sciences and the Humanities, Poznan University of Medical Sciences, 61-701 Poznan, Poland; anastazja.szula@gmail.com; 2 Phenomenological Psychopathology and Psychotherapy, Psychiatric Clinic, University of Heidelberg, 69115 Heidelberg, Germany; 3 Institute of Philosophy, Maria Curie-Skłodowska University in Lublin, 20-031 Lublin, Poland; 4 IDEAS-NCBR, 00-801 Warsaw, Poland; 5 Department of Health Sciences, University of Florence, 50121 Firenze, Italy; 6 Centro de Estudios de Fenomenologia y Psiquiatria, Diego Portales’ University, Santiago 8370067, Chile

**Keywords:** phenomenological interview, lived time, lived experience, mental disorders, patients, phenomenology, phenomenological psychopathology, quantitative phenomenology, psychiatric diagnosis, structured interview, temporality, time perception

## Abstract

Abnormal experiences of time (ATEs) are an established object of research in phenomenological psychopathology. **Objective:** The purpose of this study was the first validation of the Transdiagnostic Assessment of Temporal Experience (TATE), a structured phenomenological interview concerning ATEs in individuals with diverse mental health conditions, and its adaptation for the Polish language. **Methods:** The research employed a mixed-method approach and consisted of several phases including (1) consensual translation; (2) construct and content validation by an expert panel; (3) direct feedback from patients with lived experiences of alcohol addiction, borderline personality, autism, and clinical depression; (4) an auditorium questionnaire with 98 respondents without mental health issues, who were both interviewed and gave qualitative feedback; and (5) a final expert panel and approval. **Results:** Following multiple stages of modification, the final TATE demonstrates strong internal consistency and validity (Cronbach’s α = 0.9), with strong correlations between the frequency, intensity, and impairment of various forms of ATEs as well as their rare occurrence among healthy participants. **Conclusions:** TATE represents a multidimensional and structured quantitative phenomenological approach to temporal experience for psychiatry and clinical psychology. This article presents the validated version of TATE for Polish alongside updated administration guidelines. It is now the state-of-the-art TATE that may be further adapted to other languages, including English.

## 1. Introduction

The exploration of abnormal time experiences (ATEs) has a rich history in phenomenological research spanning over a hundred years. An abundance of research indicates that temporality is altered in psychopathological circumstances, with specific disturbances often linked to different disorders. These temporal alterations are evident in conditions such as depressive disorders [1,2,3,4,5,6,7,8,9,10,11,12,13,14,15,16,17,18,19], mania [19,20,21,22,23,24], borderline personality disorder [25,26,27,28], addictions [29,30], and others. Empirical research has also been conducted within this realm. Among the research instruments available for clinical purposes, albeit without a genuinely phenomenological foundation [31], are those focused on objectifying temporal experiences, such as the temporal Perceptual Aberration Scale [32], a tool used in neuropsychological experimental studies. There are also many psychological questionnaires aimed at giving an account of subjective temporal experience [10,33,34,35,36], such as perhaps the most widely used Zimbardo Time Perspective Inventory (ZTPI) [37], which is a five-factor survey designed to assess personal variations in time perspective profiles and specific time perspective biases. Psychometric and cognitive science research on time perception often combines tasks measuring executive functioning with ZTPI [38,39] and other inventories or rating scales [7,22,40].

On the other hand, within phenomenology, most research is non-empirical and concerned with understanding the structures of temporality and how these structures change in abnormal temporal experience [11,13,14,15,18,24,28,29,30,31,41,42,43,44,45,46,47,48,49,50,51]. There are also studies in applied phenomenology focused on researching lived experience, including the experience of time, more empirically, and these are mostly present in the clinical context [52]. Detailed semi-structured interview tools, such as EASE [53], EAFI [54], and EAWE [55], are designed to address experiences associated with schizophrenia spectrum conditions. Such tools are not commonly used in qualitative phenomenological research. Instead, interview scripts are often designed to fit the specific purposes of the study. This allows the scripts to align with the complexities of particular conditions, such as schizophrenia [56], cancer [57], major depression [58], eating disorders [59], borderline personality disorder [60], and autism [61].

Finally, more quantitative approaches to the phenomenology of temporal experience are both survey-based and experimental. For example, Mishara and Giersch [62] used an indirect phenomenological approach by interpreting the experimental findings on schizophrenia patients with the conceptual apparatus of phenomenology. Conversely, Vogel et al. [17] utilised a qualitative time questionnaire and content analysis in their study of patients suffering from major depressive disorder. Similarly, Moskalewicz et al. [57,63,64] used a sequential research design with qualitative phenomenological interviews on the lived experience of time during chemotherapy followed by front-loaded phenomenology surveys to integrate hypothesis testing with phenomenological insights.

Nevertheless, phenomenologically grounded quantitative empirical studies are scarce, especially when compared with the sheer amount of non-phenomenological research tools available. To address this issue, Stanghellini et al. [65] introduced the first fully phenomenological structured interview tool to facilitate large-scale empirical investigations of ATEs in psychopathological conditions, called the Transdiagnostic Assessment of Temporal Experiences (TATE). TATE incorporates a diverse range of questions focusing on various types of ATEs, and its quantitative nature makes it suitable for systematic empirical studies involving patients diagnosed with different disorders. To this date, however, TATE has not been applied or empirically validated.

In its design, TATE is closest to the abovementioned EASE and EAWI semi-structured phenomenological interviews but also differs in key aspects. First, TATE can be applied to a variety of experiences related to mental illness and not primarily to the schizophrenia spectrum. Second, it is focused solely on temporality. A significant portion of EAWE is also devoted to the experience of time; namely, there are 24 items that overlap in content with TATE due to the content of temporal phenomena covered. However, within the whole EAWE interview design, these items cover one of the six domains (the other five being space, other persons, language, atmosphere, and existential orientation), whereas TATE consists of 42 items distributed along seven dimensions that are all focused on temporality. EASE, on the other hand, contains only one item concerning the disturbance of temporality within the domain of cognition and stream of consciousness (with two sub-types: disturbance in subjective and existential time). Third, TATE has three layers of scoring regarding frequency, intensity, and impairment (0–7 scale), while EASE and EAWE have one (with 1–5 and 0–2 scales, respectively). Finally, while EASE assesses experiences on a lifetime basis and EAWE shorter and more focused periods, TATE, as initially intended, addresses ATEs that took place during the month preceding the interview.

This paper describes the step-by-step theoretical and empirical validation of TATE in the Polish language by a group of mental health professionals and phenomenologists prior to its application in research with participants with mental disorders. The tool underwent content and construct validation through an expert panel, empirical validation that included people with lived experiences of illness, and several modifications. The following sections of this article will refer to the subsequent phases of the validation procedure. The supplement contains an updated version of the TATE interview (TATE PL) validated in Polish for use in front-loaded phenomenological research involving people with experience of mental disorders.

## 2. Materials and Methods

### 2.1. TATE Structure

TATE consists of 42 items, each referencing a different ATE. The items are divided into seven groups representing dimensions of lived temporality (see [65] for detailed descriptions). These dimensions are (1) anomalies of synchrony, (2) anomalies of time structure, (3) anomalies of implicit time flow, (4) anomalies of explicit time flow, (5) anomalous experiences of the past, (6) anomalous experiences of the present, and (7) anomalous experiences of the future. Each item has an operational definition of the ATE it references and three prompts. The interviewee should self-assess the severity of each ATE on three scales: frequency, intensity, and the extent of impairment of daily activities. This allows for scores on a 1–7 Likert scale that quantitatively measure each item.

### 2.2. Adaptation and Validation Procedure

**Figure 1:**
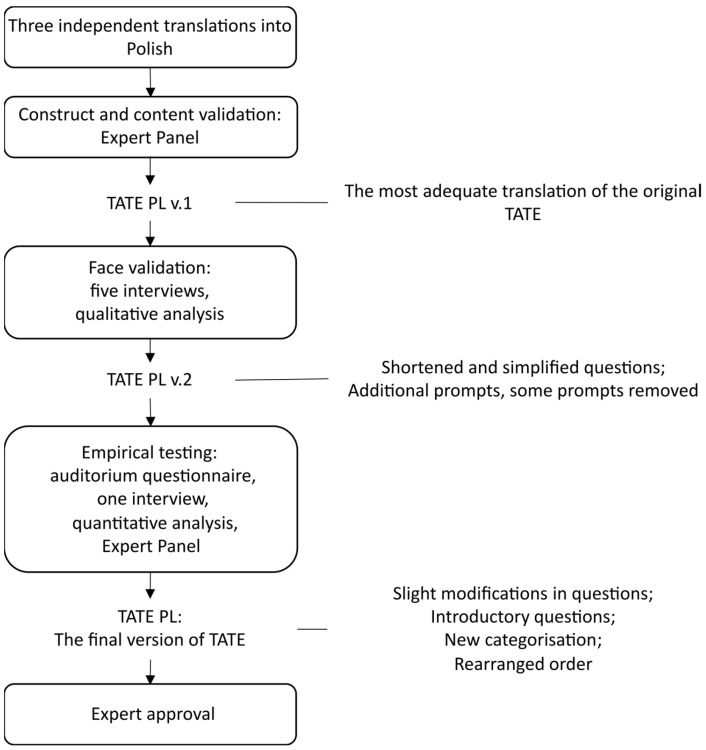
The adaptation and validation of TATE.

The process of adapting and validating TATE into Polish employed a mixed-method approach and consisted of five stages: (1) consensual translation; (2) construct and content validation by an expert panel; (3) direct feedback from patients with lived experiences of alcohol addiction, borderline personality, autism, and clinical depression; (4) an auditorium questionnaire with 98 respondents without mental health issues; and (5) a final expert panel and approval (see Figure 1 for details).

## 3. Results

### 3.1. Translation and Expert Panel

Three researchers—a student, a psychologist, and the leading researcher (the latter two trained in phenomenology)—independently translated TATE into Polish. These translations underwent scrutiny within an entirely native-speaking Polish expert panel comprising two translators, one author of the original version of TATE, and additional project-involved researchers, psychiatrists, and philosophers, totalling six experts. During the discussion, the expert panel selected each item’s most fitting translation, guided by construct and content validity criteria—ensuring the operational definitions and prompts align with their intended phenomenological meaning and aim. Consequently, this process gave rise to the first Polish version of TATE (TATE PL v.1). The double translation was not necessary since the original English TATE was never validated, as well as because the final Polish version is ultimately not identical to the original. A double translation will be necessary to adapt the now state-of-the-art Polish version into English.

### 3.2. Face Validation of TATE PL v.1

#### 3.2.1. Face Validation Procedure

The next stage involved assessing the comprehensibility of the items when presented to subjects with mental disorder diagnoses, evaluating the linguistic clarity, the conveyed meaning, and the cohesion of the tool. This assessment encompassed five individual interviews, featuring one participant without a mental disorder diagnosis and four people with lived experience of distinct conditions. We selected the sample purposively in line with future planned research and included alcohol addiction, borderline personality disorder, autism, and a mood disorder, specifically clinical depression. We also took into consideration the diversity of educational backgrounds to account for any possible misunderstanding of often sophisticated descriptions of items. Four participants were female, one male (aged 26–54); one had primary education, one secondary, and three a master’s degree.

The participants were aware of the interrelation of the definitions and prompts within each item of TATE. As proved necessary at the beginning of the first interview, the participants viewed each item displayed on a screen simultaneously as they listened to the interviewer reading it aloud. For each operational definition and each prompt in TATE, the participants received the following set of questions:

1. Do you understand the prompt?
2. Explain how you understand the prompt. What do you think the prompt says?
3. Can you reformulate it to be more understandable if you do not understand it?

At the end of the interview, the participants were asked if it had been enough to understand how they experienced time and if more atypical experiences had not been touched upon. The subsequent analysis focused on the discrepancies between the participants’ answers to the interview questions and their comments regarding each definition and prompt. The objective was to improve TATE’s comprehensibility.

#### 3.2.2. Face Validation Results

Participants encountered significant difficulties in recollecting and comprehending the definitions and prompts, necessitating the display of each on the screen. Moreover, the participants required additional time for most items to read and reread the content independently. They stated that many definitions and prompts were too long and complex to grasp the meaning upon hearing them for the first time. The interviews showed that not all prompts were harmonious with the operational definitions. In some cases, the participants interpreted the definition and prompts very differently in the sense that they related them to a different area of experience. This showed that the questionnaire is not entirely internally consistent regarding reception by subjects not trained in phenomenology. Table 1 shows differences in the interpretation on the example of item 5.d (biographical disintegration) by two participants, P2 and P4.

The analysis that followed revealed several issues with the tool. The main concern is the excessive complexity and, in some cases, imprecision of the operational definitions and prompts—the participants simply did not understand what was asked of them. The lack of understanding might indicate a particular ATE not occurring for a specific participant’s experience; however, the complexity of the definitions and prompts often led to overinterpreting. As shown in the example in Table 2, the interpretations of direct metaphors and ambiguous words frequently diverged, leading to varying understandings among participants. Often, individuals tended to interpret the prompts in ways that aligned with their diverse experiences.

Resulting modifications implemented in TATE focused on simplifying questions, eliminating direct metaphors and ambiguous words, removing redundant prompts, and introducing new prompts for the most challenging items. To prevent bias, original examples of everyday situations were removed from operational definitions, relocated to additional prompts, or placed in a new optional section for the interviewer’s reference. The General Information to the Interviewees section was shortened and simplified. Despite all that, all items of the original TATE were retained, and their order remained unchanged. The frequency, intensity, and impairment scales were altered to add a “zero” value when an ATE never occurs to better align them with one another (see Table 3).

### 3.3. Quantitative Testing of TATE PL v.2

#### 3.3.1. Quantitative Testing Procedure

The modified questions were compiled into an online questionnaire. We decided to use a convenience sample with students as participants since they are one of the most widely studied populations globally. The only exclusion criterion was a reported mental illness of any kind. The questionnaire started with the modified General Information to the Interviewees section and included all 42 TATE items in the original order. The participants were presented with the entire description of items, the operational definitions, and all the prompts so they could comment on their internal coherence. They were asked to self-assess their experiences and mark adequate responses on scales. If aparticipant marked an item as something they did not experience, they moved to the next one, omitting the questions about intensity and impairment of daily activities and scoring zero on each scale. The procedure also included meta-questions about both the phenomena represented by the items and the quality of the test. The researcher encouraged the participants to ask questions, give suggestions, and comment.

#### 3.3.2. Quantitative Testing Sample

A total of 119 Polish students from Poznan University of Medical Sciences completed the questionnaire during several auditorium sessions. We included comments from participants who reported mental disorder diagnoses in the reassessment but excluded their responses from the quantitative analysis. As a result, the sample subjected to final analysis amounts to 98 participants, 78 (79.59%) of which were female, and 20 (20.41%) male, aged between 18 and 31 years old. A total of 80 (81.63%) participants had a high school diploma, 17 (17.35%) had a bachelor’s degree or equivalent, and one (1.02%) had a master’s degree. The testing was supported by one full TATE interview with a participant without a mental disorder diagnosis, a male aged 24.

At this stage, another expert panel was organised, comprising the leading researcher, one author of the original version of TATE, and two practising interviewers, a sociologist, and a philosopher.

#### 3.3.3. Quantitative Testing Data Exploration

The quantitative assessment aimed to produce results that would enable the calculation of the reliability (Cronbach’s α) of TATE PL v.2 and provide an overview of ATEs in participants without a mental disorder diagnosis. All calculations were performed using Microsoft Excel Version 2403 and IBM SPSS Statistics 23. The figures were generated with MS Excel, SPSS, and Plotly for Python 3.12.4, and edited in Inkscape 1.0.2.

To perform comparisons, we calculated minimal, maximal, median, and mean results and interquartile ranges for each item on each scale: frequency, intensity, impairment, as well as its total severity (see Table 4). The original TATE defines the severity of ATEs as the maximum score reached in each dimension. For example, if a specific item is scored 5 for intensity, 4 for frequency, and 3 for impairment, the interviewer should retain the maximum score reached: 5 [65] (p. 82). To avoid uncertainties and align the scales for representation, we re-defined severity as the mean of scores reached in each dimension. In the example above, the severity would be equal to 4. Similarly to frequency, intensity, and impairment, each item can thus receive a summary severity score on the 0–7 scale.

**Figure 2:**
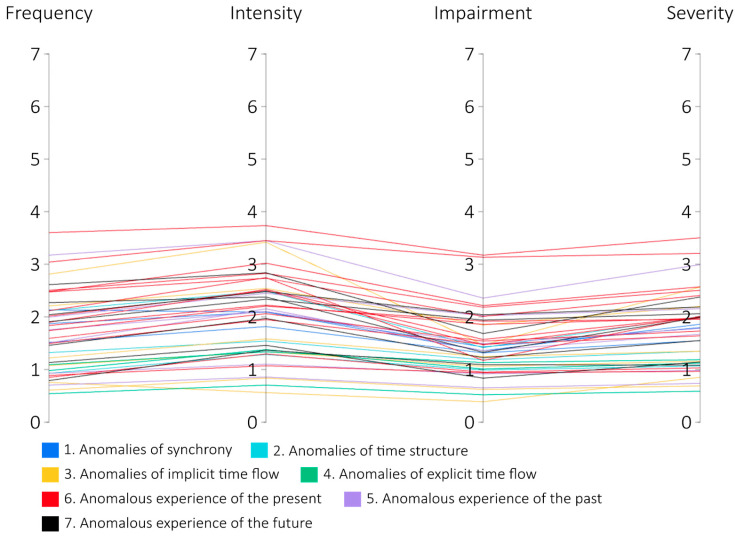
Mean scores for each TATE item across scales (N = 98).

We calculated the mean results for each TATE item on each scale (see Figure 2). There are no exceptionally high scores, with the highest mean being 3.735, recorded for item 6.j (racing against time) on the intensity scale.

**Figure 3:**
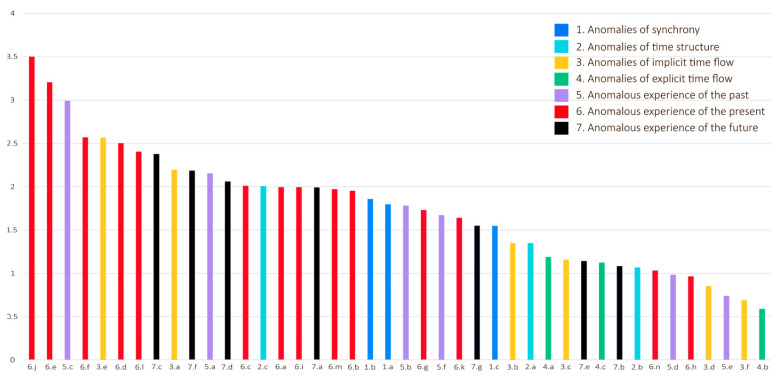
Mean severity for each TATE item, highest to lowest.

The final severity scale served as the basis for comparisons between the items (Figure 3). It is worth noting that the results in the fourth dimension, anomalies of explicit time flow, are exceptionally low. The results are otherwise evenly distributed, with item 6.j (racing against time) scoring the highest for severity.

**Figure 4:**
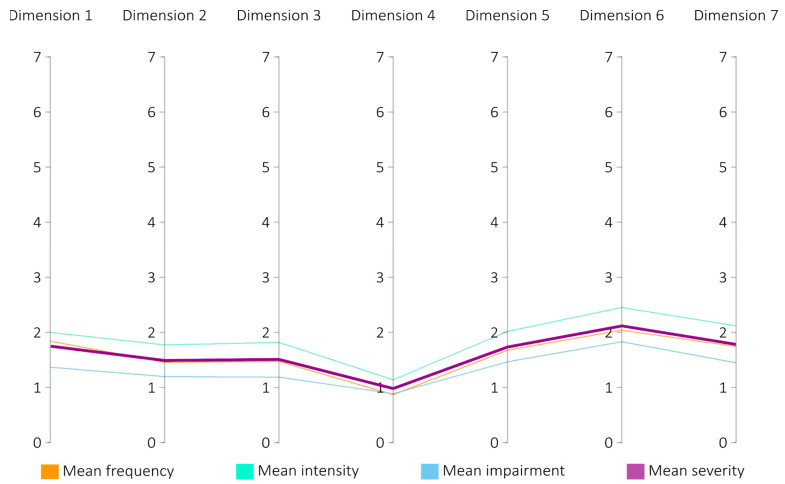
Mean scores for each TATE scale across dimensions (N = 98).

Similarly, the mean results calculated for all dimensions are rather low and do not significantly diverge across them. In all cases, the intensity scale mean values are the highest, and the impairment scale mean values are the lowest, as presented in Figure 4. Crucially, the severity scores range only between 0.966 (for anomalies of explicit time flow) and 2.107 (for anomalous experience of the present), which corresponds to the experienced phenomena appearing sometimes at most, being very mild, and presenting only a slight and hardly noticeable impairment.

#### 3.3.4. Quantitative Testing Correlations

Correlations for each dimension are presented in the Supplementary Materials.

The reliability analysis demonstrated exceptionally high internal consistency for each scale. Table 5 shows the Cronbach’s α value calculated for each TATE scale separately.

**Figure 5:**
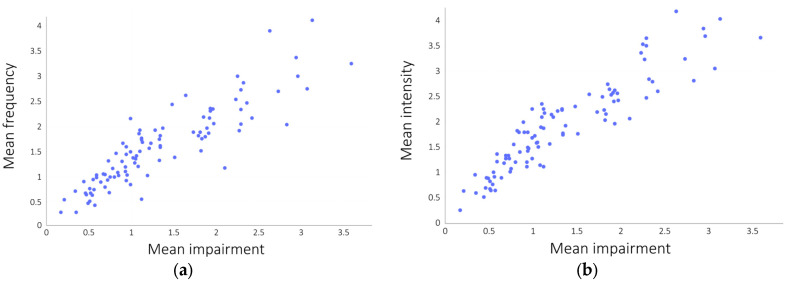
(**a**) Correlations between mean scores for frequency and impairment. (**b**) Correlations between mean scores for intensity and impairment.

We also found a high correlation between the frequency, intensity, and impairment of various forms of ATEs among participants without a mental disorder diagnosis. This is due to the fact that overall results are low, and the internal consistency of the questionnaire is very high. However, there are variations in strength. We hypothesised that intensity and impairment may correlate more strongly than frequency and impairment. This proved false for several items: in the case of 1.a, 2.b, 3.a, 3.e, 4.c, 5.e, 6.b, and 6.c, Spearman’s ρ for frequency and impairment is higher than Spearman’s ρ for intensity and impairment. However, when considering mean results calculated for each dimension, Spearman’s ρ for mean frequency and impairment is always smaller than Spearman’s ρ for mean intensity and impairment. Therefore, the hypothesis proved true for entire dimensions, albeit with slight differences in ρ. The outcome is similar when calculating the correlation coefficient for the mean values of all the results, as shown in Figure 5. We may hypothesise that these results would largely differ in populations with mental illness, where, for example, very disturbing experiences may take place less frequently, or very intense experiences might not be considered harmful. In this context, the current result may be considered a benchmark for future comparisons.

The main conclusion at this stage is that ATEs are hardly present in participants without mental illness diagnoses. There are individual differences, but overall, the results indicate a low severity of ATEs, regardless of the temporal dimension addressed.

### 3.4. Further Modifications of TATE PL

#### 3.4.1. Premises

Based on all gathered expert and participant feedback and results, we introduced the last and most significant improvements to the tool. Semi-structured phenomenological interviews are designed with a particular disorder in mind (e.g., [53,54,55] for schizophrenia). The questions are open-ended, and they allow the interviewees to respond freely and with little obstruction. They also allow the interviewers to adjust the script to a particular interview situation. Interviewers consider the interviewees’ capabilities and how they communicate (e.g., [61] for autism). After collecting the answers, researchers analyse and categorise the responses based on pre-written operational definitions. Such freedom is impossible to achieve in a structured interview set when generating quantitative data. Additionally, being a transdiagnostic tool, TATE’s framework cannot be modified to answer the needs of particular participants. Nevertheless, some adjustments were necessary. Most modifications were introduced so that the interview would be easy to administer by researchers and clinicians without a phenomenological background and more understandable and approachable for the interviewees. To do this, we drew inspiration from more typical ways of conducting a phenomenological interview while retaining the structured and quantitative character of the script.

#### 3.4.2. New Introduction

An introduction to any interview should focus on making the interviewee interested and involved. The interviewer should begin the exchange with a guiding question to begin building a relationship with the interviewee. With time, they should relax and concentrate closely on what the other is sharing [66]. Presenting the participant with a lengthy introduction at the beginning of the interview risks them losing focus and interest. Therefore, we propose removing the General Information to the Interviewees section from the original TATE and replacing it with open-ended questions regarding everyday experiences and time flow. This way, we not only create an opportunity for the interviewee to engage in the conversation actively but also guide their focus towards contemplation of their temporal experiences.

The first question prompts the interviewee to discuss their perception of time, with the responses serving as a reference for subsequent TATE questions.

1. How, normally, does time pass for you?

Alternatively: how, normally, do your days pass?

A person who seldom contemplates time and their temporal experiences might face challenges delving deeply into the topic of ATEs. The second proposed question estimates the interviewee’s inclination towards considering these aspects.

2. How important is time in your life?

Alternatively: how often do you think about time?

#### 3.4.3. Rearrangement of Items

During the live interviews and through expert recommendations, it became clear that the organisation of TATE items needs revamping. This is because trust building and flow (necessary in any interview script) were not considered in the original order of items. TATE is an interview tool that concerns experiences related to mental illness and well-being, and, as the patients made clear through their feedback, the interview might act as a trigger. Additionally, asking challenging or emotionally sensitive questions too early before establishing trust disrupts the progression and may cause unease [66]. For these reasons, we decided to reorganise the interview script to create an atmosphere of trust that would facilitate accessing the phenomenal experiences of interest by both interlocutors.

The division of TATE into seven dimensions (as presented in Table 4) was created a priori in accordance with the phenomenological distinctions regarding the structures of temporal experience. Nevertheless, we conducted a factor analysis based on severity values to check for any underlying patterns, and it yielded a one-factor solution accounting for 30% of the total variance and with mean factor loadings of 0.543 for all 42 items (min–max 0.351–0.667). No factors aligning with the seven dimensions were found, but it was not expected since the theoretical phenomenological framework was on principle organising the items in a front-loaded phenomenological manner (which is why the division into dimensions was kept). Also, this could be accounted for by the generally low frequency, intensity, and impairment of the temporal phenomena measured, which is due to the character of the sample. We may, however, speculate that, in this case, the latent variable represents the underlying structure of normal temporal experience. Further research with clinical populations should explore this issue in more detail.

To reorganise the items in terms of facilitating the interview flow, we identified the most understandable category of narrative or biographical temporality. Prioritising present experiences as our reference point, we also addressed past and future experiences as being most intuitively comprehended by participants. The next step involved organising all items into threads, ensuring each element in a given thread corresponded to the preceding one. Subsequently, we created new sets of items based on these threads.

It is important to note that these sets do not constitute an interpretative conceptual framework but only a conversational one aiming to enhance communication. Since data obtained with TATE are quantitative and do not include personal accounts, we propose summarising the interview with a supplementary, open-ended question. The qualitative material may help gain additional insights into lived time and be potentially used to further refine the tool.

The new conversational sets are as follows:

1. Routine (6.l, 6.i, 6.a)—the items in this set are related to the everyday passage of time, allowing the interviewer to refer to the answers for the introductory questions;
2. Plans (7.c, 6.b, 6.g);
3. Goals and responsibilities (6.e, 6.d, 6.j, 6.f, 3.e, 6.c, 7.a);
4. Memories (5.c, 5.f, 5.e);
5. Deceleration (6.m, 3.b, 3.f, 3.d);
6. Acceleration (3.a, 4.c);
7. Disintegration (3.c, 2.a, 2.b, 4.a, 4.b, 6.n, 6.h, 5.d);
8. Confusion (1.b, 1.c, 1.a, 2.c, 6.k);
9. Negative temporal feelings (5.a, 5.b, 7.b, 7.d, 7.g);
10. Alertness (7.f, 7.e);
  + Open-ended question.

As mentioned above, the original phenomenological categorisation into seven dimensions was not abandoned and should still guide the items’ arrangement for post hoc phenomenological analysis.

#### 3.4.4. Revision of Items

During the validation process, we observed participants struggling to recall the beginning of an item description as it approached its end. Participants required multiple repetitions and the opportunity to independently read the definitions and prompts to retain all information and be able to formulate a response. In the original TATE, most operational definitions are lengthy and refer to phenomena that are not immediately accessible. As a result, a TATE interview is rather challenging for the interviewee; it requires them to learn, understand, and apply each definition to their often previously unnamed experiences as the interview goes on.

To enhance the accessibility of the script, we consolidated the operational definition with the first prompt in each item, resulting in a significant reduction in length. This modification aimed to minimise the number of words interviewees must remember and process. To maintain the complexity and specificity of each item, some of the content removed during this process was transferred to additional questions.

Another significant challenge stemmed from the lack of precision of certain items due to the excessive use of direct metaphors (categorised by Steen [67]) and broad, context-dependent terms like “things”, “fate”, etc. Discussing time without resorting to metaphors is inherently demanding, given the abstract nature of the concept in human experience. Nevertheless, relying solely on metaphors can lead to varied participant interpretations, contributing to imprecision.

In response, we adopted a two-step approach. First, we aimed to describe each phenomenon in the most straightforward and direct manner in the main question. Subsequently, we introduced alternative, more intricate, or metaphorical explanations in additional questions. This approach allowed us to use metaphors as supplementary tools to enhance comprehension after presenting the core information straightforwardly. It is crucial to note that a lack of understanding of a specific item may suggest the absence of that particular experience in the interviewee. It is not necessary for all interviewees to grasp every item perfectly.

Some items required more significant changes than others. The most noteworthy cases are items 1.a (desynchronisation with objective time) and 5.b (nostalgia). In 1.a, the original TATE asks about the disorientation in time and losing grip on the measure of years, months, weeks, days, or hours. As stated by multiple participants, losing grip on the measure of hours (and, for some participants, days) is a frequent issue, drastically different in their experience from disorientation about what week, month, or year it is. We concluded that item 1.a might refer to several different phenomena and opted for the removal of “hours” and “days” from it. As for item 5.b, we classified it as suited for the negative temporal experiences set. However, initially, it focuses on the positive idea of one’s past, in contrast to what happens in the present. To fit the item into the set, we opted to redirect the focus onto the negative experience of the present, in contrast to the past. However, we kept the prompts focusing on the strong nostalgic sentiments towards the past in the additional questions.

#### 3.4.5. Expert Approval

The results gathered from the questionnaire, the interviews, and the expert suggestions served as a basis for the third and final version of TATE PL. All forty-two items targeting particular temporal phenomena listed in the original TATE remained in this version, albeit with the abovementioned modifications. After completion, TATE PL underwent a final expert evaluation by two philosophers, one of whom a practising interviewer, and was approved for research purposes.

## 4. Discussion

### 4.1. Summary of the Validation Procedure

TATE is a phenomenological interview script and analysis tool encompassing a wide range of phenomena related to time experience. At the outset of the validation process, we translated the script into Polish and discussed it with an expert panel. Subsequently, we conducted several interviews to establish its face validity and found out that it proved overly complex for participants with no phenomenological background. Following this, the simplified version of the script underwent additional empirical testing on a group of students and conceptual assessment with another expert panel. We found that all kinds of ATEs occur rarely in healthy participants, or they are only rarely recognised. The results and suggestions received during the validation process served as the foundation for the implemented modifications. These changes encompass revising and reorganising the items and replacing the General Information for the Interviewees with a series of introductory questions. To get richer data, we included an open-ended question at the script’s conclusion. The original categorisation of items into seven dimensions remains for the purposes of analysing the data. The proposed Polish version of the interview script will be applied in research involving participants with diverse mental conditions grouped by diagnosis. The results should not only outline the ATEs based on pre-established categories but also potentially show any cross-diagnostic overlaps. The script may undergo further improvements when that research is complete.

### 4.2. Punishment Problem

The length and complexity of TATE can make interviewees hesitant to give affirmative answers, fearing it might prolong the session. As pointed out by Jensen et al. [68], interviewees may discover that acknowledging a symptom leads to more questions. If they simply deny having a particular symptom, the interview structure bypasses additional related questions, potentially making the interview shorter. To prevent such issues and ensure high-quality data representing the lived phenomena of participants not trained in phenomenology, we suggest that the interviewers actively engage the interviewees in the conversation about their temporal experiences.

### 4.3. Administration of TATE PL

The interviewer will briefly explain the interview’s purpose and then proceed with introductory questions. Afterwards, they will move on to the items; they will read the main question and, in instances of prolonged pauses or confusion, proceed to the supplementary prompts, further rephrasing them if necessary. The interviewer is allowed to provide examples of particular experiences that could imply the presence of a particular ATE, but it is advised to avoid doing so in order not to bias the interviewee. TATE, as initially intended, assesses ATEs that took place during the previous month. Interviewees might have trouble framing their time experiences in a one-month perspective; therefore, they should be reminded and guided frequently. The answers should all be marked on the provided Likert scales, either by the interviewer in consultation with the interviewee or—preferably—by the interviewee themself. Visual representation of such scales can help the interviewee refer to the parameters. The whole interview is planned for about an hour, but it might take longer, depending on the interviewee’s needs. The script describes all instructions in detail (see Supplementary Materials).

### 4.4. Enhancement of Phenomenological Research Tools

The validation process has yielded several significant insights, particularly concerning the interviewing experience, including participant feedback and the development of an improved script. These insights offer valuable suggestions for empirical phenomenologists in general. One crucial observation underscores the importance of the questions’ order in a structured phenomenological interview because the participants’ understanding of meaning is affected by how questions are arranged and the larger narrative structure of the whole interview. Organising questions according to a common-sense understanding that resonates with the participants is more effective than solely relying on the researcher’s theoretical–phenomenological framework. Similarly, item descriptions are understood much better and more consistently among interviewees when they avoid often obscure philosophical terms.

A broader conclusion is the need for empirical phenomenological research tools to aim for accessibility for those without a formal background in phenomenology or lacking in philosophical, reflective capacities. Applying a phenomenological interview effectively requires collaborative efforts involving participants, experts by experience, and researchers with formal training. This cooperative approach ensures that the interview tools are relevant and user-friendly, enhancing the overall quality and impact of applied phenomenological research.

### 4.5. Broader Significance

Psychology and psychopathology aim to identify traits or states that can characterise individuals, identify their fragilities, and possibly predict their behaviour. Among these, the best known are cognitive functioning (e.g., capacities and metacognition), defence mechanisms (e.g., projection and denial), and early warning signs (e.g., mild experiences of depersonalisation/derealisation). Phenomenology points to other parameters; among those, the central one is how each person experiences time. Some people live exclusively in the present as if there was no tomorrow; others cannot help but always regret the past or feel remorse; others live constantly in preparation for the future, trying to foresee its adversities and anticipate the appropriate counter-moves to protect themselves. Literature offers a wealth of examples of the characterisation of types of human existence based on temporal experience; emblematic is the fable of the cicada and the ant, in which these two forms of life are represented either as living in an absolute present (the cicada) or in the constant, laborious effort to accumulate resources for the uncertain future. 

The contribution of our study, which aims to provide a reliable and effective tool for identifying how people experience time, is to enrich the clinician’s toolbox with an advanced phenomenological tool for characterising personality, in addition to the better-known ones mentioned above.

### 4.6. Conclusions

The way of experiencing time is the fingerprint—or one of the leading indices—of the way of being in the world of human persons. As such, a better understanding of it can contribute substantially to psychological and psychopathological knowledge. TATE represents a multidimensional and structured quantitative phenomenological approach to temporal experience for psychiatry and clinical psychology. The TATE PL interview script is currently the state-of-the-art version of TATE that may be further adapted to other languages, including English.

## Figures and Tables

**Table 1:** Face validation: internal incoherence within an item.

Item 5.d Biographical Disintegration	Comments Supplied by Participants P2 and P4 (Translated from Polish)
In some cases, one may have the feeling that the most important life events are disconnected, as if the thread that keeps them together were missing. Does something like that happen to you?	[P2] *Hardly understandable, I’m not sure what it is about, the most important events could be a wedding, a child, an illness,* etc.*—they are disconnected, what does aunt X’s divorce have to do with uncle Z’s death, if these events concern me only then there is a unifying thread, and if they involve the same person—*e.g., *marriage to Y, child to Y, divorce to Y. The question is not very specific.*
	[P4] *I understand that there are milestones in life and there is no continuity of events.*
Do you feel that the choices, activities, jobs, and interests that you have experienced throughout your life are unrelated to each other, as if they were about different people?	[P2] *Yes, I’ve realised recently—I lived my life not the way I wanted because I did not know how to live otherwise, I could not express desires, needs, that something is not right,* etc. *Childhood programming—it’s not mine, behaviour different from me, it’s the way I was bred, imprinted.*
	[P4] *If I feel that some of my hobbies and job are very different from each other.*
Does it seem to you that the most important events of your past have happened a little at random, as if there were no history that connects them?	[P2] *There are no coincidences, everything that happens, happens not by accident, although we perceive some situations as accidental. The question is complicated, not quite sure what it is about, not very specific.*
	[P4] *If I feel that I have random events.*

**Table 2:** Face validation: problematic wording example.

Item 4.b Time Reversal, Prompt 3	Comments Supplied by Participants P1–P5 (Translated from Polish)
Do you feel that the things around you are going back to their starting point?	[P1] *If I feel as if the situation, despite my efforts to solve it, returns to the same place where it started.*
	[P2] *What does “things” mean? Is it phenomena, people or rather things—clothes? More probably phenomena, events. Weird question.*
	[P3] *When you throw something and it bounces back, it comes back to you.*
	[P4] *I understand it in a more positive sense, that an everyday routine reappears after a stressful time.*
	[P5] *I don’t know what things around me, what I could connect it all with, what might be going back.*

**Table 3:** Modified ATE rating scales.

Frequency	Intensity	Impairment
0. It never happens.	0. -	0. -
1. Rarely.	1. I don’t feel it at all.	1. It does not interfere with what I do.
2. Sometimes.	2. It’s very mild.	2. Slight interference, I almost don’t notice it.
3. Often.	3. It’s mild but it’s there.	3. Slight interference but I can still manage with everyday activities.
4. Quite often.	4. It’s pretty strong.	4. It starts to interfere with my everyday activities.
5. Very often.	5. It’s strong.	5. It interferes much with most of my everyday activities.
6. Nearly always.	6. It’s very strong.	6. It interferes very much with almost all my everyday activities.
7. It’s always like this.	7. It’s unbearably strong.	7. It interferes extremely with all my everyday activities.

**Table 4:** Descriptive statistics (N = 98).

ATE Dimension	Item	Frequency Mean (Me; Min; Max; IQR)	Intensity Mean (Me; Min; Max; IQR)	Impairment Mean (Me; Min; Max; IQR)	Severity Mean (Me; Min; Max; IQR)
1. Anomalies of synchrony	1.a	1.872 (2.0; 0; 6; 1.0)	2.092 (2.0; 0; 6; 2.0)	1.429 (1.0; 0; 4; 1.0)	1.798 (1.7; 0; 5.0; 1.0)
	1.b	2.133 (2.0; 0; 6; 1.0)	2.092 (2.0, 0, 6, 2.0)	1.357 (1.0; 0; 4; 1.0)	1.861 (1.8; 0; 5.0; 1.8)
	1.c	1.515 (1.0; 0; 7; 2.0)	1.816 (2.0; 0; 6; 3.0)	1.316 (1.0; 0; 6; 2.0)	1.549 (1.7, 0; 6.3; 2.3)
	Summary	1.840 (2.0; 0; 7; 2.0)	2.000 (2.0; 0; 6; 2.0)	1.367 (1.0; 0; 6; 1.0)	1.736 (1.7; 0; 6.3; 1.7)
2. Anomalies of time structure	2.a	1.321 (1.0; 0; 6; 2.0)	1.541 (1.0; 0; 7; 3.0)	1.184 (1.0; 0; 6; 2.0)	1.349 (1.3; 0; 6.3; 2.3)
	2.b	0.929 (0.5; 0; 6; 1.3)	1.286 (0.5; 0; 6; 2.0)	0.990 (0.5; 0; 7; 2.0)	1.068 (0.5; 0; 6.3; 1.8)
	2.c	2.117 (2.0; 0; 7; 2.0)	2.490 (3.0; 0; 5; 1.0)	1.418 (1.0; 0; 4; 1.0)	2.009 (2.0; 0; 4.7; 1.5)
	Summary	1.456 (1.0; 0; 7; 2.0)	1.772 (2.0; 0; 7; 3.0)	1.197 (1.0; 0; 7; 2.0)	1.475 (1.3; 0; 6.3; 2.3)
3. Anomalies of implicit time flow	3.a	1.209 (0.0; 0; 7; 2.0)	2.541 (3.0; 0; 7; 3.0)	1.847 (1.0; 0; 7; 2.0)	2.199 (2.0; 0; 7.0; 2.3)
	3.b	2.119 (1.0; 0; 6; 2.0)	1.582 (1.0; 0; 6; 3.0)	1.245 (1.0; 0; 6; 2.0)	1.349 (1.3; 0; 5.7; 2.3)
	3.c	1.077 (0.0; 0; 7; 2.0)	1.337 (0.0; 0; 6; 2.0)	1.061 (0.0; 0; 6; 2.0)	1.158 (0.0; 0; 6.3; 2.0)
	3.d	0.757 (0.0; 0; 7; 1.0)	1.013 (0.0; 0; 7; 2.0)	0.789 (0.0; 0; 6; 1.0)	0.853 (0.0; 0; 6.0; 1.6)
	3.e	2.811 (2.0; 0; 7; 2.5)	3.418 (4.0; 0; 7; 3.0)	1.469 (1.0; 0; 6; 1.0)	2.566 (2.3; 0; 6.3; 1.8)
	3.f	0.607 (0.0; 0; 4.5; 1.0)	0.837 (0.0; 0; 5; 2.0)	0.622 (0.0; 0; 5; 1.0)	0.689 (0.0; 0; 4.5; 1.3)
	Summary	1.473 (1.0; 0; 7; 2.0)	1.818 (2.0; 0; 7; 3.0)	1.187 (1.0; 0; 7; 2.0)	1.493 (1.3; 0; 7.0; 2.3)
4. Anomalies of explicit time flow	4.a	1.092 (0.0; 0; 7; 2.0)	1.337 (0.0; 0; 7; 3.0)	1.133 (0.0; 0; 7; 2.0)	1.187 (0.0; 0; 7.0; 2.3)
	4.b	0.541 (0.0; 0; 6; 1.0)	0.704 (0.0; 0; 5; 1.0)	0.520 (0.0; 0; 4; 1.0)	0.558 (0.0; 0; 4.5; 1.1)
	4.c	0.980 (0.5; 0; 6; 2.0)	1.378 (0.5; 0; 5; 3.0)	1.010 (0.5; 0; 5; 2.0)	1.122 (0.5; 0; 5.3; 2.0)
	Summary	0.871 (0.0; 0; 7; 1.0)	1.139 (0.0; 0; 7; 2.0)	0.888 (0.0; 0; 7; 1.0)	0.966 (0.0; 0; 7.0; 1.7)
5. Anomalous experience of the past	5.a	1.995 (2.0; 0; 7; 3.0)	2.449 (2.0; 0; 7; 4.0)	2.020 (2.0; 0; 7; 3.0)	2.155 (2.0; 0; 7.0; 3.5)
	5.b	1.755 (1.0; 0; 7; 3.0)	2.102 (2.0; 0; 7; 3.0)	1.490 (1.0; 0; 7; 2.0)	1.782 (1.7; 0; 6.0; 2.7)
	5.c	3.173 (3.0; 0; 7; 2.5)	3.449 (3.0; 0; 7; 3.0)	2.357 (2.0; 0; 7; 3.0)	2.993 (3.0; 0; 7.0; 2.7)
	5.d	0.929 (0.0; 0; 7; 2.0)	1.102 (0.0; 0; 7; 2.0)	0.918 (0.0; 0; 5; 2.0)	0.983 (0.0; 0; 6.3; 1.7)
	5.e	0.704 (0.0; 0; 6; 1.0)	0.857 (0.0; 0; 5; 2.0)	0.653 (0.0; 0; 5; 1.0)	0.738 (0.0; 0; 4.7; 1.3)
	5.f	1.505 (1.0; 0; 7; 2.0)	2.153 (2.0; 0; 7; 4.0)	1.357 (1.0; 0; 6; 2.0)	1.672 (1.5; 0; 6.3; 2.4)
	Summary	1.677 (1.0; 0; 7; 2.0)	2.019 (2.0; 0; 7; 3.0)	1.466 (1.0; 0; 7; 2.8)	1.721 (1.3; 0; 7.0; 2.7)
6. Anomalous experience of the present	6.a	1.898 (1.5; 0; 7; 2.3)	2.520 (2.0; 0; 7; 4.0)	1.571 (1.0; 0; 7; 2.3)	1.997 (2.0; 0; 7.0; 3.0)
	6.b	1.740 (1.0; 0; 7; 3.0)	2.204 (2.0; 0; 7; 4.0)	1.918 (2.0; 0; 7; 3.0)	1.954 (1.8; 0; 6.7; 3.0)
	6.c	2.117 (2.0; 0; 7; 2.0)	2.745 (3.0; 0; 6; 2.0)	1.173 (1.0; 0; 4; 0.0)	2.012 (2.0; 0; 4.7; 1.4)
	6.d	2.505 (2.0; 0; 7; 2.0)	2.827 (2.0; 0; 7; 3.0)	2.184 (2.0; 0; 6; 2.0)	2.505 (2.3; 0; 6.3; 2.2)
	6.e	3.041 (3.0; 0; 7; 3.5)	3.449 (3.0; 0; 7; 1.0)	3.133 (3.0; 0; 7; 2.0)	3.207 (3.0; 0; 7.0; 2.4)
	6.f	2.480 (2.0; 0; 7; 2.4)	3.020 (3.0; 0; 7; 2.0)	2.214 (2.0; 0; 6; 2.0)	2.571 (2.7; 0; 6.3; 1.8)
	6.g	1.592 (1.0; 0; 7; 2.0)	2.061 (2.0; 0; 6; 3.0)	1.541 (1.0; 0; 6; 2.0)	1.731 (1.7; 0; 5.3; 2.7)
	6.h	0.888 (0.0; 0; 7; 1.0)	1.071 (0.0; 0; 7; 2.0)	0.939 (0.0; 0; 7; 2.0)	0.966 (0.0; 0; 7.0; 1.7)
	6.i	2.015 (2.0; 0; 7; 2.0)	2.500 (2.0; 0; 7; 3.0)	1.469 (1.0; 0; 7; 1.0)	1.995 (1.8; 0; 7.0; 1.5)
	6.j	3.602 (3.0; 0; 7; 4.0)	3.735 (4.0; 0; 7; 2.3)	3.173 (3.0; 0; 7; 2.0)	3.503 (3.4; 0; 7.0; 3.0)
	6.k	1.495 (1.0; 0; 6; 2.0)	1.949 (2.0; 0; 7; 3.0)	1.480 (1.0; 0; 6; 2.3)	1.641 (1.3; 0; 5.3; 2.7)
	6.l	2.474 (2.0; 0; 7; 2.4)	2.735 2.0; 0; 7; 3.0)	2.010 (2.0; 0; 7; 2.0)	2.406 (2.3; 0; 7.0; 2.8)
	6.m	1.837 (1.5; 0; 7; 2.0)	2.224 (2.0; 0; 6; 3.3)	1.857 (2.0; 0; 6; 3.0)	1.973 (1.7; 0; 6.3; 3.0)
	6.n	0.852 (0.0; 0; 7; 1.0)	1.296 (0.0; 0; 6; 3.0)	0.949 (0.0; 0; 6; 1.3)	1.032 (0.0; 0; 5.2; 2.0)
	Summary	2.038 (2.0; 0; 7; 3.0)	2.448 (2.0; 0; 7; 4.0)	1.829 (1.0; 0; 7; 3.0)	2.107 (2.0; 0; 7.0; 3.3)
7. Anomalous experience of the future	7.a	2.270 (2.0; 0; 7; 3.0)	2.378 (2.0; 0; 7; 4.0)	1.327 (1.0; 0; 6; 2.0)	1.991 (2.0; 0; 5.3; 3.0)
	7.b	0.791 (0.0; 0; 7; 1.0)	1.367 (0.0; 0; 7; 3.0)	1.092 (0.0; 0; 7; 2.0)	1.083 (0.0; 0; 6.3; 2.0)
	7.c	2.612 (2.0; 0; 7; 3.5)	2.837 (3.0; 0; 7; 2.0)	1.684 (1.0; 0; 5; 2.0)	2.378 (2.3; 0; 5.7; 2.0)
	7.d	1.908 (1.0; 0; 7; 3.0)	2.337 (2.0; 0; 7; 4.0)	1.939 (2.0; 0; 7; 3.0)	2.061 (1.7; 0; 7.0; 3.3)
	7.e	1.133 (1.0; 0; 7; 2.0)	1.459 (1.0; 0; 7; 2.0)	0.837 (1.0; 0; 6; 1.0)	1.143 (1.0; 0; 6.3; 2.0)
	7.f	2.041 (2.0; 0; 6; 3.0)	2.480 (2.0; 0; 6; 4.0)	2.041 (2.0; 0; 6; 3.0)	2.187 (2.0; 0; 5.7; 3.7)
	7.g	1.459 (1.0; 0; 7; 2.0)	1.969 (2.0; 0; 7; 3.0)	1.224 (1.0; 0; 6; 2.0)	1.551 (1.5; 0; 6.3; 2.3)
	Summary	1.745 (1.0; 0; 7; 2.0)	2.118 (2.0; 0; 7; 3.0)	1.449 (1.0; 0; 7; 2.0)	1.771 (1.7; 0; 7.0; 2.8)

**Table 5:** Reliability analysis for each TATE scale.

Scale	Cronbach’s α, N = 98
Frequency	0.916
Intensity	0.936
Impairment	0.947

## Data Availability

Raw data are attached as Supplementary Material.

## Supplementary Materials

The following supporting information can be downloaded at: https://www.mdpi.com/article/10.3390/jcm13154325/s1, TATE PL Interview Script; Raw Data; TATE Auditorium Questionnaire Data Calculations.
