# Supplementary material for: Transdiagnostic Assessment of Temporal Experience (TATE) in Mental Disorders—Empirical Validation and Adaptation of a Structured Phenomenological Interview
Source: J Clin Med. 2024 Jul 24;13(15):4325. doi: 10.3390/jcm13154325 (PMC11313341; doi:10.3390/jcm13154325)
Supplement: Supplementary file 1 [file jcm-13-04325-s001.zip › MDPI TATE sup 1.pdf]

Published as a Supplementary Material to: A. Szuła, M. Moskalewicz, G. Stanghellini. Transdiagnostic Assessment of Temporal Experience (TATE) in Mental Disorders – Empirical Validation and Adaptation of a Structured Phenomenological Interview.

TATE PL Interview Script.

TATE PL Scenariusz wywiadu.

Uwaga: wykluczone powinny być osoby w kryzysie suicydalnym. Koniecznie skonsultować tę kwestię z personelem przed umówieniem wywiadu.

Instrukcja: wywiad rozpoczyna się od przedstawienia się przez osobę prowadzącą, opisania warunków i wyrażenia zgody na udział.

Jestem [osoba prowadząca informuje kim jest i gdzie pracuje].

Chciałabym przeprowadzić dziś z tobą wywiad ustrukturyzowany na temat doświadczenia czasu. Potrwa on nie więcej niż półtora godziny. Wyniki będą analizowane zbiorczo, a badanie jest anonimowe, nie rejestrujemy imienia i nazwiska badanego/ną.

Czy wyrażasz zgodę na udział w wywiadzie?

Instrukcja: pierwsza seria pytań metryczkowych:

Wiek:

Płeć:

Poziom wykształcenia:

Czy badany/a pracuje:

W jakim wymiarze:

W jakiej strukturze:

Instrukcja: Rozpoczynając wywiad, osoba prowadząca zadaje badanemu/nej kilka pytań wprowadzających, aby zachęcić badanego/ą do nawiązania konwersacji oraz by otworzyć temat doświadczenia czasu. Pytania wprowadzające:

Najpierw, powiedz mi, proszę…

Jak, normalnie, płynie dla ciebie czas?
Alternatywnie: jak, normalnie, mijają ci dni?

Na ile czas jest ważny w twoim życiu?
Alternatywnie: jak często myślisz o czasie?

Instrukcja: Odpowiedzi na pytania wprowadzające nie muszą być analizowane, ale mogą służyć jako uzupełnienie do właściwej części wywiadu. Wprowadzenie powinno trwać nie więcej niż kilka minut, podczas których **mówić będzie przede wszystkim badany/a**. Po krótkiej konwersacji następuje wprowadzenie do tematyki wywiadu i przedstawienie warunków uczestnictwa:

Teraz przejdę do serii konkretnych pytań na temat tego, jakie jest twoje doświadczenie czasu. Niektóre pytania będą brzmiały podobnie do siebie, postaraj się jednak odpowiedzieć na każde.

Odpowiedzi na poszczególne pytania są dobrowolne, zawsze możesz odmówić odpowiedzi.

Jeśli czegoś nie zrozumiesz lub nie będziesz pewien/a, o co chodzi w pytaniu, powiedz mi o tym.

Interesuje mnie twoje doświadczenie [zdiagnozowane zaburzenie].
LUB
Szczególnie ważne są twoje doświadczenia z ostatniego miesiąca.

Odpowiadając na pytania, zapiszesz swoje odpowiedzi na skalach.

Instrukcja: Scenariusz wywiadu składa się z 42 elementów, które składają się z jednego pytania głównego oraz pytań pomocniczych. Odpowiedź na każde z pytań zostanie zanotowana w postaci ilościowej na podanych niżej skalach. Podczas wywiadu osoba prowadząca skorzysta z ekranu komputera lub z kart papierowych, prezentujących pytanie główne i skale (patrz załącznik 1).

Instrukcja: Osoba prowadząca posłuży się następującymi skalami częstości, intensywności i uporczywości podczas wywiadu. Badany/a wskazuje odpowiedź sam, na ekranie lub na papierowej karcie, lub podaje odpowiedź osobie prowadzącej. W drugim przypadku osoba prowadząca zaznacza odpowiedź tak, aby Badany/a widział ekran lub kartę i zaznaczoną odpowiedź. Osoba prowadząca, w razie potrzeby, dopytuje, by móc zaznaczyć odpowiednią liczbę na skali do analizy ilościowej.

CZĘSTOŚĆ		INTENSYWNOŚĆ		UPORCZYWOŚĆ	
Jak często występuje?		Jak bardzo jest intensywne?		Jak bardzo przeszkadza w codziennych czynnościach?	
0	Nigdy się nie zdarza.	0	-	0	-
1	Rzadko.	1	W ogóle nieodczuwalne.	1	Nie przeszkadza.
2	Czasami.	2	Bardzo łagodne.	2	Trochę przeszkadza, ale prawie tego nie zauważam.
3	Dość często.	3	Łagodne, ale odczuwalne.	3	Trochę przeszkadza, ale mimo to radzę sobie z codziennymi czynnościami.
4	Często.	4	Dość silne.	4	Zaczyna przeszkadzać mi w codziennych czynnościach.
5	Bardzo często	5	Silne.	5	Przeszkadza mi w większości codziennych czynności.
6	Prawie cały czas.	6	Bardzo silne.	6	Bardzo przeszkadza mi w prawie wszystkich codziennych czynnościach.
7	Cały czas.	7	Nieznośnie silne.	7	Bardzo przeszkadza mi we wszystkich codziennych czynnościach.

Instrukcja: Osoba prowadząca zadane główne pytanie, a następnie, w razie niezrozumienia lub potrzeby pogłębienia, zadaje pytania pomocnicze. Może wybrać, które pytania pomocnicze zada i w jakiej kolejności. Badany/a powinien mieć swobodę udzielenia otwartej odpowiedzi na każde pytanie. **Końcowa odpowiedź, wyrażona liczbowo na skalach, zawsze udzielona będzie wobec pytania głównego**.

Instrukcja: Jeśli Badany/a twierdzi, że czegoś w ogóle nie doświadcza, nie wie, z czym miałby to skojarzyć, lub w ogóle nie rozumie pytanie, osoba prowadząca zaznacza odpowiedź „Nigdy się nie zdarza" na skali częstości i przechodzi do następnego pytania.

Instrukcja: Osoba prowadząca, przy każdym elemencie, podaje badanemu/nej nową kartę lub wyświetla kolejne pytanie na ekranie.

Instrukcja: Osoba prowadząca, po każdym pytaniu, na które Badany/a udzielił odpowiedzi, określa postrzegany stopień współbrzmienia badanego/ną z pytaniem – ocenia, na ile Badany/a zrozumiał pytanie. Kryterium rozumienia jest zgodność pojęciowa pytania i odpowiedzi do niego oraz zgodność pomiędzy afektem badanego/ną a treścią odpowiedzi. W tym celu może skorzystać z poniższej skali:

1	Badany/a nie zrozumiał treści pytania, jego/jej odpowiedź i afekt nie są zgodne.
2	Trudno powiedzieć, czy Badany/a zrozumiał pytanie i czy jego/jej odpowiedź i afekt są zgodne.
3	Badany/a zrozumiał treść pytania i jego/jej afekt jest zgodny z odpowiedzią.

ZESTAW: RUTYNA

Pytanie 1., element 6.l.
Czy zdarza ci się preferować swoje istniejące przyzwyczajenia wobec nowości i zmian?

PYTANIA POMOCNICZE:
Gdybyś mógł wybrać, co byś wybrał: rutynę dnia codziennego czy zmiany?
Zastanów się, czy...
...nie lubisz zmian w swoim życiu?
...masz niekiedy wrażenie, że każda zmiana może wzbudzić w tobie niepewność, a nawet nieść zagrożenie lub niebezpieczeństwo?
...obawiasz się zmian w życiu codziennym, bo mogą to być zmiany na gorsze?

Pytanie 2., element 6.i.
Czy zdarza ci się poszukiwać silnych, niecodziennych doświadczeń, aby wyrwać się z codziennej rutyny?

PYTANIA POMOCNICZE:
Co pomaga ci się wyrwać z codziennej rutyny? Czy to musi być silne, intensywne i niecodzienne?
Zastanów się, czy...
…aby poczuć się w pełni żywym/ą potrzebujesz niezwykle silnych i intensywnych doznań?

Pytanie 3., element 6.a.
Czy zdarza ci się potrzebować intensywnej przyjemności, aby poczuć, że żyjesz?

PYTANIA POMOCNICZE:
Kiedy czujesz, że naprawdę żyjesz?
Zastanów się, czy...
...starasz się ekscytować każdym dniem tak, jakby miał być ostatnim?
...wolisz natychmiastową przyjemność, niż odroczoną?
...w celu osiągnięcia przyjemności możesz zignorować ryzyko, jakie się z tym wiąże?

ZESTAW: PLANY

Pytanie 4., element 7.c.
Czy zdarza ci się bardzo szczegółowo planować i projektować przyszłość?

PYTANIA POMOCNICZE:
Jak często planujesz? Czego dotyczą twoje plany? Jak bardzo są szczegółowe?
Zastanów się, czy...
...masz niekiedy wrażenie, że bez dobrej organizacji przyszłości czuł/abyś się niekomfortowo?
...poprzez szczegółową organizację czasu próbujesz zapobiegać wszelkim problemom, które mogą wystąpić?

Pytanie 5., element 6.b.
Czy zdarza ci się mieć wrażenie, że nie masz żadnego wpływu na to, co będzie?

PYTANIA POMOCNICZE:
Zastanów się, czy...
…czujesz się kowalem swojego losu, czy raczej nie?
...odczuwasz brak wpływu na swoje własne działania?
...odczuwasz brak wpływu na nadchodzące wydarzenia?
...zdarza ci się myśleć, że sprawy potoczą się tak, jak się potoczą i nie ma sensu próbować ich zmieniać?
...masz niekiedy wrażenie, że Twoim życiem kieruje jakaś wyższa siła, los?

Pytanie 6., element 6.g.
Czy uważasz, że życie składa się z tego, co tu i teraz, więc zdarza ci się ignorować to, co długoterminowe?

PYTANIA POMOCNICZE:
Jak bardzo koncentrujesz uwagę na teraźniejszości, na tym co aktualnie się dzieje w twoim życiu?
Zastanów się, czy...
…zdarza ci się, że tak bardzo koncentrujesz się na tym, co tu i teraz, że przestajesz myśleć o przyszłości?
...masz niekiedy wrażenie, że często zmieniasz przyjaciół, partnerów, zainteresowania, pracę, przyjemności i tym podobne?
...nie robisz długoterminowych planów?
...nie interesuje cię, jakie będą Twoje relacje z ludźmi w odległej przyszłości?
...nie zastanawiasz się, gdzie będziesz pracować w przyszłości?
...nie poświęcasz się długoterminowym zainteresowaniom i hobby?
...masz niekiedy wrażenie, że Twoje życie składa się z momentów, z których każdy jest zupełnie inny?

ZESTAW: CELE I OBOWIĄZKI

Pytanie 7., element 6.e
Czy zdarza ci się unikać swoich obowiązków, odkładać je na przyszłość?

PYTANIA POMOCNICZE: Zastanów się, czy...
...masz tendencję do odkładania nieprzyjemnych bądź trudnych zadań na później?
...masz niekiedy wrażenie, że na wszystko wystarczy ci czasu?

Pytanie 8., element 6.d.
Czy zdarza ci się nie kończyć zadań bo masz poczucie, że trzeba je poprawić, udoskonalić?

PYTANIA POMOCNICZE: Zastanów się, czy...
...odczuwasz potrzebę wielokrotnego sprawdzania swojej pracy, wykonywanych przez siebie czynności?
...OBAWIASZ SIĘ, że z tym, co robisz, może być coś nie tak i wolał/abyś to sprawdzić jeszcze raz?
…NIE CHCESZ kończyć zadań, ponieważ wolał/abyś do nich wrócić i sprawdzić je jeszcze raz?
...zdarza ci się myśleć, że wszystko można zrobić lepiej, więc wolał/abyś pozostawiać zadania niedokończone, by móc je w przyszłości poprawić?

Pytanie 9., element 6.j.
Czy zdarza ci się odczuwać, że masz za mało czasu, aby zdążyć ze wszystkim, co masz do zrobienia?

PYTANIA POMOCNICZE: Zastanów się, czy...
...masz niekiedy poczucie „ścigania się z czasem”?
...masz niekiedy wrażenie, że bezradnie zostajesz w tyle wobec swoich zobowiązań?
... często sprawdzasz godzinę, aby wiedzieć dokładnie ile czasu minęło i ile zostało na zrobienie tego, co w danym momencie robisz?

Pytanie 10., element 6.f.
Czy zdarza ci się odczuwać potrzebę jak najszybszego wykonania zadań, ponieważ w przeciwnym razie będziesz odczuwać przykry stres?

PYTANIA POMOCNICZE: Zastanów się, czy...
…obawiasz się, że jeśli nie rozwiążesz wszystkich problemów od razu, coś może pójść nie tak?
...masz tendencję do natychmiastowego kończenia wszystkich zadań, bo czujesz, że jeśli je odłożysz, będziesz ciągle o nich myśleć?
...chciał/abyś czasem jak najszybciej wykonać zadanie, bo praca nad nim cię stresuje?

Pytanie 11., element 3.e.
Czy zdarza ci się zrywać się do działania, czuć wielką potrzebę, by natychmiast działać, natychmiast realizować cele?

PYTANIA POMOCNICZE: Zastanów się, czy...
...zdarza ci się mieć nagłe poczucie, że musisz działać, zająć się czymś, wziąć się do roboty?
...zdarza ci się mieć nagłe poczucie, że czujesz się silniejszy/a niż kiedykolwiek wcześniej?
...zdarza ci się czuć, że jesteś w stanie pokonać wszystkie przeszkody stojące na drodze do Twoich celów?

Pytanie 12., element 6.c.
Czy zdarza ci się czuć energię i pewność siebie tak wielką, jakbyś mógł/mogła naraz wiele zrobić?

PYTANIA POMOCNICZE:
Zastanów się, czy...
...masz niekiedy wrażenie, że tu i teraz możesz wykonać ogromną liczbę zadań?
...niekiedy czujesz, jakbyś miał/a zdolność zrobienia naraz tysiąca różnych rzeczy?
...masz niekiedy wrażenie, że możesz natychmiast zrobić wszystko, o czym pomyślisz?
…zdarza ci się czuć, że mógłbyś przenosić góry?

Pytanie 13., element 7.a.
Czy zdarza ci się postrzegać przyszłość jako niemal nieskończone źródło możliwości, nowości i szans, po które musisz sięgnąć?

PYTANIA POMOCNICZE: Zastanów się, czy...
...przyszłość wydaje ci się wypełniona wydarzeniami, których nie należy przegapić?
...zdarza ci się odczuwać całkowitą pewność co do tego, co ma nadejść?

ZESTAW: WSPOMNIENIA

Pytanie 14., element 5.c.
Czy odczuwasz silną tendencję do analizowania historii swojego życia?

PYTANIA POMOCNICZE: Zastanów się, czy...
...ciągle wracasz do wydarzeń z przeszłości i rozważasz je na nowo?
...spędzasz dużo czasu na rozważaniu minionych wydarzeń?

Pytanie 15., element 5.f.
Czy zdarza ci się nagłe wspomnienia przeżywać z taką intensywnością, jakby przeszłość działa się teraz?

PYTANIA POMOCNICZE: Zastanów się, czy...

...miewasz nagłe, intensywne wspomnienia, które mocno przeżywasz?
...przeżywasz minione wydarzenia z taką samą siłą, jak wtedy, gdy się odbywały?

Pytanie 16., element 5.e.
Czy zdarza ci się mieć wrażenie, że niektóre z twoich wspomnień nie należą do ciebie?

PYTANIA POMOCNICZE: Zastanów się, czy...
...masz niekiedy wrażenie, że niektóre historie z twojego życia nie dotyczą ciebie, jakby przydarzyły się komuś innemu?
...masz niekiedy wrażenie, że jakieś wydarzenie lub sytuacja, w której brałeś udział w przeszłości, nie zdarzyła się na prawdę?

ZESTAW: SPOWOLNIENIE

Pytanie 17., element 6.m.
Czy zdarza ci się mieć wrażenie, że nic się nie dzieje, twoje dni są monotonne i wydają się nigdy nie mijać?

PYTANIA POMOCNICZE: Zastanów się, czy...
...masz niekiedy wrażenie, że godziny płyną wolno i ciągną się?
...masz niekiedy wrażenie, że w twoich dniach nic się nie dzieje?
…wydaje ci się, że twoje życie to nieustająca monotonia?

Pytanie 18 ., element 3.b.
Czy zdarza ci się mieć wrażenie, że czas płynie wolniej, jakby to co robisz miało się nigdy nie skończyć – i nie potrafisz wytłumaczyć, czemu?

PYTANIA POMOCNICZE: Zastanów się, czy...
...wydaje ci się, że to co robisz, czego doświadczasz, nigdy się nie skończy, choć nie wiesz, dlaczego?
...wydaje ci się, że godziny lub dni trwają znacznie dłużej niż zwykle, wloką się?

Pytanie 19., element 3.f.
Czy zdarza ci się mieć wrażenie, że czas zatrzymuje się gwałtownie bądź stopniowo – i nie potrafisz wytłumaczyć, czemu?

PYTANIA POMOCNICZE: Zastanów się, czy...
...zdarza ci się mieć poczucie, że wszystko się zatrzymało?
...zdarza ci się mieć poczucie, że czas zatrzymał się, jakby miał już nigdy nie ruszyć?

Pytanie 20., element 3.d.

Czy zdarza ci się mieć wrażenie, że choć czas wokół ciebie wciąż płynie, w tobie przestał?

PYTANIA POMOCNICZE: Zastanów się, czy...
…zdarza ci się mieć poczucie, że wszystko się w Tobie zatrzymało, że wewnątrz ciebie czas przestał płynąć?
...zdarza ci się mieć wrażenie, że jesteś poza czasem, a świat „dzieje się" nadal?

ZESTAW: PRZYSPIESZENIE

Pytanie 21., element 3.a.
Czy zdarza ci się mieć wrażenie, że czas płynie szybciej – i nie potrafisz wytłumaczyć, czemu?

POMOCNICZE PRZYKŁADY DLA PROWADZĄCEGO WYWIAD:
wszystko dzieje się szybciej, niż zwykle, ale nie wiesz dlaczego, nie wiesz, jaka jest przyczyna

Pytanie 22., element 4.c.
Czy zdarza ci się mieć wrażenie, jakbyś nagle przeskoczył/a do przodu w czasie?

Instrukcja: Pamiętaj! W tym pytaniu nie uwzględnia się snu lub stanu pod wpływem środków psychoaktywnych.

PYTANIA POMOCNICZE: Zastanów się, czy...
…zdarza ci się mieć poczucie, że coś nagle przerzuca cię do przyszłości?
…zdarza ci się mieć poczucie, że nie było jakiegoś odcinka czasu?
…zdarza ci się mieć poczucie, że jesteś gwałtownie przerzucony/a w przyszłość?
…zdarza ci się mieć poczucie, że nagle przeskoczyłeś/aś w przyszłość?
…zdarza ci się mieć poczucie, że przerzucił cię w przyszłość wehikuł czasu?

ZESTAW: DEZINTEGRACJA

Pytanie 23., element 3.c.
Czy zdarza ci się mieć wrażenie, że czas upływa w zmiennym tempie, przyspiesza i zwalnia – i nie potrafisz tego wytłumaczyć?

PYTANIA POMOCNICZE: Zastanów się, czy...
...zdarza ci się czuć zdezorientowanym/ą zmiennym tempem czasu?
...zdarza ci się czuć zdezorientowanym/ą zmienną prędkością upływu czasu?

Pytanie 24., element 2.a.
Czy zdarza ci się mieć wrażenie, że czas biegnie skokowo, że zatrzymuje się i ponownie rozpędza?

PYTANIA POMOCNICZE: Zastanów się, czy...
… masz poczucie że zdarzenia są oderwane od siebie w czasie?

…masz poczucie, że rzeczy, wydarzenia, pojawiają się ni stąd, ni zowąd?
...wydaje ci się, że każda chwila jest osobna, niezwiązana z innymi chwilami?
...wydaje ci się, że ruch rzeczy, zwierząt, ludzi..., który obserwujesz, jest przerywany, choć powinien być ciągły?
… rzeczy i ludzie jawią się jako seria niezależnych od siebie obrazów lub migawek – nie tworzą wspólnie filmu, ale każda migawka istnieje oddzielnie?

Pytanie 25., element 2.b.
Czy zdarza ci się mieć dziwne wrażenie, że przepływ czasu zostaje przerwany przez coś nagłego lub obcego?

PYTANIA POMOCNICZE: Zastanów się, czy...
...masz niekiedy wrażenie, że upływ czasu jest nagle przerwany przez coś nietypowego?
...masz niekiedy wrażenie, że coś z zewnątrz wdziera się w twoje poczucie czasu?

Pytanie 26., element 4.a.
Czy zdarza ci się mieć wrażenie, że czas się zapętlił?

PYTANIA POMOCNICZE: Zastanów się, czy...
...wydaje ci się, że czas kręci się w kółko?
...wydaje ci się, że wciąż znajdujesz się w tych samych sytuacjach?
...zdarza ci się mieć poczucie, że ciągle robisz to samo – twoje działania ciągle zaczynają się od początku?

Pytanie 27., element 4.b.
Czy zdarza ci się mieć wrażenie, że czas się cofa?

PYTANIA POMOCNICZE: Zastanów się, czy...
...zdarza ci się mieć wrażenie, że robisz rzeczy, które już zrobiłeś/aś, jakbyś cofnął/ęła się w czasie?

Pytanie 28., element 6.n.
Czy zdarza ci się czuć, jakby coś w tobie mogło przyspieszyć lub spowolnić upływ czasu?

PYTANIA POMOCNICZE: Zastanów się, czy...
...zdarza ci się odczuwać niecierpliwość tak wielką, że zmienia ona bieg czasu?
...zdarza ci się czuć napięcie wewnętrzne, które zmienia upływ godzin?
...zdarza ci się czuć, jakby „buzowało” w tobie coś, co może zmienić bieg czasu?

Pytanie 29., element 6.h.

Czy zdarza ci się mieć wrażenie, że w tym, co doświadczasz, brakuje elementu, który wszystko łączy w całość?

PYTANIA POMOCNICZE: Zastanów się, czy...
...masz niekiedy wrażenie, że to, co przeżywasz jest niespójne, jakby Twoim przeżyciom brakowało „centrum", „jądra", pewnej myśli przewodniej?
...masz niekiedy wrażenie, że brakuje „nici" łączącej ze sobą to, czego doświadczasz?
...masz niekiedy wrażenie, że rzeczy dzieją się przypadkowo, w sposób niepowiązany ze sobą?
...masz niekiedy wrażenie, że życie wydaje ci się chaosem pozornie niepowiązanych ze sobą doświadczeń?

Pytanie 30., element 5.d.

Czy zdarza ci się mieć wrażenie, że różne aspekty twojego życia są rozłączone, niepowiązane, jakby nie należały do jednej – twojej – historii?

PYTANIA POMOCNICZE: Zastanów się, czy...
...masz niekiedy wrażenie, że Twoje wybory, aktywności, praca i zainteresowania, nie są ze sobą powiązane, jakby należały do różnych osób?
...różne wydarzenia z Twojej przeszłości wydarzyły się trochę przypadkowo, jakby nie łączyła ich żadna historia?

ZESTAW: ZAGUBIENIE

Pytanie 31., element 1.b.
Czy, patrząc na innych ludzi, wydarzenia, przedmioty, zdarza ci się mieć wrażenie, że każde z nich ma swój własny upływ czasu?

PYTANIA POMOCNICZE: Zastanów się, czy...
…zdarza ci się mieć wrażenie, że czas płynie w innym tempie dla jednych ludzi, w innym dla drugich ludzi, w innym dla różnych przedmiotów, w innym dla różnych wydarzeń?
...wydaje ci się, że czas płynie dla różnych ludzi w różnym tempie? (Nawet jeśli WIESZ, że płynie w tym samym)
...wydaje ci się, że wszystko wokół ciebie dzieje się osobno, choć powinno dziać się razem?
...rzeczy dzieją się w nieskoordynowany sposób, choć powinny być skoordynowane?
...wydaje ci się, że czas płynie dla każdego człowieka, każdej rzeczy i każdego wydarzenia inaczej?
…wydaje ci się, że brakuje uzgodnienia pomiędzy upływem czasu, w którym żyje każdy człowiek, istnieje rzecz, dzieje się wydarzenie?

POMOCNICZE PRZYKŁADY DLA PROWADZĄCEGO WYWIAD:
na przykład sposób, w jaki ludzie się poruszają lub rozmawiają, stał się niespójny i dziwny;
na przykład ludzie na ulicy stoją lub poruszają jedni bez związku z innymi, jakby każde z nich robiło coś oddzielnie, bez odniesienia do innych

Pytanie 32., element 1.c.
Czy zdarza ci się mieć wrażenie, że twój czas płynie z inną prędkością niż czas innych?

PYTANIA POMOCNICZE: Zastanów się, czy...
...wydaje ci się, że upływ czasu jest dla ciebie szybszy, niż dla otaczających cię w danej chwili ludzi?
...wydaje ci się, że upływ czasu jest dla ciebie wolniejszy, niż dla otaczających cię w danej chwili ludzi?
…czujesz, że jesteś niezsynchronizowany/a z codziennymi wydarzeniami?

POMOCNICZE PRZYKŁADY DLA PROWADZĄCEGO WYWIAD:
twój czas biegnie inaczej niż czas innych w danej chwili, na przykład twój czas biegnie inaczej niż czas nadjeżdżającego samochodu, przez co nie potrafisz przejść przez ulicę;
masz wrażenie, że jesteś niezsynchronizowany/a z innymi ludźmi, na przykład masz wrażenie, że inni ludzie myślą lub poruszają się w ślimaczym tempie w porównaniu do ciebie; lub odwrotnie straciłeś/aś kontakt ze światem, wszystko dzieje się w innym tempie niż twoje

Pytanie 33., element 1.a.
Czy zdarza ci się tracić kontrolę nad tym, który jest rok czy miesiąc?

PYTANIA POMOCNICZE: Zastanów się, czy...
...często zerkasz do kalendarza, żeby sprawdzić jaki jest rok lub miesiąc?
...zdarza się, że zapominasz ile masz lat i trudno ci to określić lub obliczyć?

Pytanie 34., element 2.c.
Czy zdarza ci się mieć wrażenie, że widziałeś/aś już miejsca, których w rzeczywistości wcześniej nie widziałeś/aś, przeżyłeś/aś wydarzenia, których w rzeczywistości wcześniej nie przeżyłeś/aś lub spotkałeś/aś ludzi, którzy w rzeczywistości są zupełnie nowi?

POMOCNICZE PRZYKŁADY DLA PROWADZĄCEGO WYWIAD:
Czy zdarza ci się spotykać ludzi, co do których masz wrażenie, że poznałeś/aś ich już w przeszłości;
że byłeś/aś już w miejscach, które w rzeczywistości widzisz po raz pierwszy;
że doświadczyłeś/aś już zdarzeń lub sytuacji, które tak naprawdę są dla ciebie nowe

Pytanie 35., element 6.k.
Czy zdarza ci się być tak zaabsorbowanym/ą sobą i swoimi myślami, że jesteś zaskoczony/a tym, co się dzieje wokół ciebie?

PYTANIA POMOCNICZE: Zastanów się, czy...
…masz niekiedy wrażenie, że wszystko co się dzieje jest dla ciebie niespodzianką?
...zdarza ci się, że jesteś zdumiony/a tym, co cię spotyka, jako czymś niespodziewanym?

ZESTAW: NEGATYWNE ODCZUCIA TEMPORALNE

Pytanie 36., element 5.a.
Czy masz wrażenie, że, w ogólnym rozrachunku, twoja przeszłość była negatywna?

PYTANIA POMOCNICZE: Zastanów się, czy...
...nie możesz przestać myśleć o dawnych przeciwnościach losu?
...masz wrażenie, że wszystkie wcześniejsze błędy lub złe decyzje nadal wpływają na twoje życie?

Pytanie 37., element 5.b.
Czy zdarza ci się mieć wrażenie, że teraźniejszość jest dużo gorsza od przeszłości?

PYTANIA POMOCNICZE: Zastanów się, czy...
...ciągle wracasz myślami do minionych chwil, jakby to był jakiś złoty wiek, inny od teraźniejszości?
...czujesz ogromną tęsknotę za tym, co minione?
...zdarza ci się mieć wrażenie, że wszystko co dobre się skończyło?

Pytanie 38., element 7.b.
Czy zdarza ci się mieć wrażenie, że wszystko, co cię miało spotkać, już się zdarzyło i nie ma już dla ciebie przyszłości?

PYTANIA POMOCNICZE: Zastanów się, czy...
...nie są już potrzebne ci nadzieje, pragnienie i cele, ponieważ dla ciebie wszystko się już skończyło?
...wydaje ci się, że koniec jest blisko?

Pytanie 39., element 7.d.
Czy zdarza ci się obawiać, że twoje życie będzie się tylko pogarszać?

PYTANIA POMOCNICZE: Zastanów się, czy...
...zamartwiasz się tym, co może się wydarzyć?
...obawiasz się, że w przyszłości czekają cię same kłopoty i nieprzyjemności?
...masz niekiedy wrażenie, że w przyszłości spotka cię coś bardzo przykrego?

Pytanie 40., element 7.g.
Czy zdarza ci się poszukiwać okazji do rewanżu na tych, którzy cię skrzywdzili?

PYTANIA POMOCNICZE: Zastanów się, czy...
...zdarza ci się czuć wielką złość na to, co ci się przytrafia, przez co chcesz się odegrać?
...czujesz, że nie jesteś traktowany/a sprawiedliwie, więc szukasz sposobu na wyrównanie rachunków?
...zdarza ci się czekać na odpowiedni moment, żeby pokazać wszystkim, że jesteś kimś więcej niż tym, za kogo cię mają?

ZESTAW: CZUJNOŚĆ

Pytanie 41., element 7.f.
Czy zdarza ci się mieć wrażenie, że musisz zachować czujność, bo może zdarzyć się coś bardzo złego?

PYTANIA POMOCNICZE: Zastanów się, czy...
...odczuwasz napięcie, niepokój, bo coś złego może się nagle stać?
...obawiasz się, że coś złego się stało, a Ty zaraz się o tym dowiesz?

POMOCNICZE PRZYKŁADY DLA PROWADZĄCEGO WYWIAD:
Czy obawiasz się złych wieści, kiedy odbierasz telefon lub email?

Pytanie 42., element 7.e.
Czy zdarza ci się mieć poczucie, że wkrótce będziesz mieć wpływ na niezwykłe wydarzenia, choć nikt o tym nie wie?

PYTANIA POMOCNICZE: Zastanów się, czy...
...masz niekiedy wrażenie, że wkrótce zostanie ci powierzona szczególna misja lub zadanie do wykonania, takie, które powierza się nielicznym lub tylko tobie?
...wydaje ci się, że wkrótce wydarzy się coś wyjątkowego, czego nikt nie zauważył, a co wpłynie na wszystkich, a szczególnie na ciebie?

Czy chciał/abyś wrócić do któregoś z pytań?

Published as a Supplementary Material to: A. Szuła, M. Moskalewicz, G. Stanghellini. Transdiagnostic Assessment of Temporal Experience (TATE) in Mental Disorders – Empirical Validation and Adaptation of a Structured Phenomenological Interview.

PYTANIE OTWARTE

Opowiedz mi teraz proszę: jaki jest twój czas?

Instrukcja: końcowe pytania metryczkowe – starać się zadawać w trakcie wywiadu i notować, w razie konieczności zadać na koniec i/lub zapytać lekarza prowadzącego (szczególnie o kody ICD chorób i typ przyjmowanych leków):

Kiedy był/aś zdiagnozowany/a?

Jak długo jesteś w terapii?

Jaki jest rodzaj twojej terapii?

Z jaką regularnością odbywasz sesje?

Czy przyjmujesz leki?

Jakie leki? Jakiego typu?

Czy masz jakieś inne diagnozy zaburzeń lub chorób? Jakie?

KONIEC WYWIADU

Czy chciał/abyś jeszcze coś dodać?
