# Supplementary material for: Transdiagnostic Assessment of Temporal Experience (TATE) in Mental Disorders—Empirical Validation and Adaptation of a Structured Phenomenological Interview
Source: J Clin Med. 2024 Jul 24;13(15):4325. doi: 10.3390/jcm13154325 (PMC11313341; doi:10.3390/jcm13154325)
Supplement: Supplementary file 1 [file jcm-13-04325-s001.zip › MDPI TATE sup 3.pdf]

Published as a Supplementary Material to: A. Szuła, M. Moskalewicz, G. Stanghellini. Transdiagnostic Assessment of Temporal Experience (TATE) in Mental Disorders – Empirical Validation and Adaptation of a Structured Phenomenological Interview.

TATE auditorium questionnaire data calculations.

Table S1. Correlation matrix for general frequency, intensity, and impairment in dimension 1. Anomalies of synchrony.

| | | frequency 1 | intensity 1 | impairment 1 |
|---|---|---|---|---|
| frequency 1 | Spearman's ρ | 1.000 | 0.854** | 0.722** |
| | p-value | | 0.000 | 0.000 |
| intensity 1 | Spearman's ρ | 0.854** | 1.000 | 0.791** |
| | p-value | 0.000 | | 0.000 |
| impairment 1 | Spearman's ρ | 0.722** | 0.791** | 1.000 |
| | p-value | 0.000 | 0.000 | |

**. Correlation is significant at the 0.01 level (2-tailed)

Table S2. Correlation matrix for each item’s severity in dimension 1. Anomalies of synchrony.

| | | 1.a | 1.b | 1.c |
|---|---|---|---|---|
| 1.a | Spearman's ρ | 1.000 | 0.237* | 0.268** |
| | p-value | | 0.019 | 0.008 |
| 1.b | Spearman's ρ | 0.237* | 1.000 | 0.774** |
| | p-value | 0.019 | | 0.000 |
| 1.c | Spearman's ρ | 0.268** | 0.774** | 1.000 |
| | p-value | 0.008 | 0.000 | |

*. Correlation is significant at the 0.05 level (2-tailed)

**. Correlation is significant at the 0.01 level (2-tailed)

Table S3. Correlation matrix for general frequency, intensity, and impairment in dimension 2. Anomalies of time structure.

| | | frequency 2 | intensity 2 | impairment 2 |
|---|---|---|---|---|
| frequency 2 | Spearman's ρ | 1.000 | 0.903** | 0.846** |
| | p-value | | 0.000 | 0.000 |
| intensity 2 | Spearman's ρ | 0.903** | 1.000 | 0.875** |
| | p-value | 0.000 | | 0.000 |
| impairment 2 | Spearman's ρ | 0.846** | 0.875** | 1.000 |
| | p-value | 0.000 | .000 | |

**. Correlation is significant at the 0.01 level (2-tailed)

Table S4. Correlation matrix for each item’s severity in dimension 2. Anomalies of time structure.

| | | 2.a | 2.b | 2.c |
|---|---|---|---|---|
| 2.a | Spearman's ρ | 1.000 | 0.525** | 0.326** |
| | p-value | | 0.000 | 0.001 |
| 2.b | Spearman's ρ | 0.525** | 1.000 | 0.490** |
| | p-value | 0.000 | | 0.000 |
| 2.c | Spearman's ρ | 0.326** | 0.490** | 1.000 |
| | p-value | 0.001 | 0.000 | |

**. Correlation is significant at the 0.01 level (2-tailed)

Table S5. Correlation matrix for general frequency, intensity, and impairment in dimension 3. Anomalies of implicit time flow.

| | | frequency 3 | intensity 3 | impairment 3 |
|---|---|---|---|---|
| frequency 3 | Spearman's ρ | 1.000 | 0.943** | 0.886** |
| | p-value | | 0.000 | 0.000 |
| intensity 3 | Spearman's ρ | 0.943** | 1.000 | 0.886** |
| | p-value | 0.000 | | 0.000 |
| impairment 3 | Spearman's ρ | 0.886** | 0.886** | 1.000 |
| | p-value | 0.000 | 0.000 | |

**. Correlation is significant at the 0.01 level (2-tailed)

Table S6. Correlation matrix for each item's severity in dimension 3. Anomalies of implicit time flow.

| | | 3.a | 3.b | 3.c | 3.d | 3.e | 3.f |
|---|---|---|---|---|---|---|---|
| 3.a | Spearman's ρ | 1.000 | 0.391** | 0.513** | 0.409** | 0.244* | 0.454** |
| | p-value | | 0.000 | 0.000 | 0.000 | 0.016 | 0.000 |
| 3.b | Spearman's ρ | 0.391** | 1.000 | 0.594** | 0.289* | 0.338** | 0.475** |
| | p-value | 0.000 | | 0.000 | 0.011 | 0.001 | 0.000 |
| 3.c | Spearman's ρ | 0.513** | 0.594** | 1.000 | 0.450** | 0.371** | 0.600** |
| | p-value | 0.000 | 0.000 | | 0.000 | 0.000 | 0.000 |
| 3.d | Spearman's ρ | 0.409** | 0.289* | 0.450** | 1.000 | 0.276* | 0.431** |
| | p-value | 0.000 | 0.011 | 0.000 | | 0.016 | 0.000 |
| 3.e | Spearman's ρ | 0.244* | 0.338** | 0.371** | 0.276* | 1.000 | 0.364** |
| | p-value | 0.016 | 0.001 | 0.000 | 0.016 | | 0.000 |
| 3.f | Spearman's ρ | 0.454** | 0.475** | 0.600** | 0.431** | 0.364** | 1.000 |
| | p-value | 0.000 | 0.000 | 0.000 | 0.000 | 0.000 | |

*. Correlation is significant at the 0.05 level (2-tailed)

**. Correlation is significant at the 0.01 level (2-tailed)

Table S7. Correlation matrix for general frequency, intensity, and impairment in dimension 4. Anomalies of explicit time flow.

| | | frequency 4 | intensity 4 | impairment 4 |
|---|---|---|---|---|
| frequency 4 | Spearman's ρ | 1.000 | 0.966** | 0.962** |
| | p-value | | 0.000 | 0.000 |
| intensity 4 | Spearman's ρ | 0.966** | 1.000 | 0.965** |
| | p-value | 0.000 | | 0.000 |
| impairment 4 | Spearman's ρ | 0.962** | 0.965** | 1.000 |
| | p-value | 0.000 | 0.000 | |

**. Correlation is significant at the 0.01 level (2-tailed)

Table S8. Correlation matrix for each item's severity in dimension 4. Anomalies of explicit time flow.

| | | 4.a | 4.b | 4.c |
|---|---|---|---|---|
| 4.a | Spearman's ρ | 1.000 | 0.382** | 0.275** |
| | p-value | | 0.000 | 0.006 |
| 4.b | Spearman's ρ | 0.382** | 1.000 | 0.216* |
| | p-value | 0.000 | | 0.033 |
| 4.c | Spearman's ρ | 0.275** | 0.216* | 1.000 |
| | p-value | 0.006 | 0.033 | |

*. Correlation is significant at the 0.05 level (2-tailed)

**. Correlation is significant at the 0.01 level (2-tailed)

Table S9. Correlation matrix for general frequency, intensity, and impairment in dimension 5. Anomalous experience of the past.

| | | frequency 5 | intensity 5 | impairment 5 |
|---|---|---|---|---|
| frequency 5 | Spearman's ρ | 1.000 | 0.937** | 0.898** |
| | p-value | | 0.000 | 0.000 |
| intensity 5 | Spearman's ρ | 0.937** | 1.000 | 0.927** |
| | p-value | 0.000 | | 0.000 |
| impairment 5 | Spearman's ρ | 0.898** | 0.927** | 1.000 |
| | p-value | 0.000 | 0.000 | |

**. Correlation is significant at the 0.01 level (2-tailed)

Table S10. Correlation matrix for each item's severity in dimension 5. Anomalous experience of the past.

| | | 5.a | 5.b | 5.c | 5.d | 5.e | 5.f |
|---|---|---|---|---|---|---|---|
| 5.a | Spearman's ρ | 1.000 | 0.392** | 0.423** | 0.373** | 0.286** | 0.309** |
| | p-value | | 0.000 | 0.000 | 0.000 | 0.004 | 0.002 |
| 5.b | Spearman's ρ | 0.392** | 1.000 | 0.485** | 0.244* | 0.204* | 0.268** |
| | p-value | 0.000 | | 0.000 | 0.016 | 0.044 | 0.008 |
| 5.c | Spearman's ρ | 0.423** | 0.485** | 1.000 | 0.403** | 0.303** | 0.544** |
| | p-value | 0.000 | 0.000 | | 0.000 | 0.002 | 0.000 |
| 5.d | Spearman's ρ | 0.373** | 0.244* | 0.403** | 1.000 | 0.505** | 0.385** |
| | p-value | 0.000 | 0.016 | 0.000 | | 0.000 | 0.000 |
| 5.e | Spearman's ρ | 0.286** | 0.204* | 0.303** | 0.505** | 1.000 | 0.424** |
| | p-value | 0.004 | 0.044 | 0.002 | 0.000 | | 0.000 |
| 5.f | Spearman's ρ | 0.309** | 0.268** | 0.544** | 0.385** | 0.424** | 1.000 |
| | p-value | 0.002 | 0.008 | 0.000 | 0.000 | 0.000 | |

*. Correlation is significant at the 0.05 level (2-tailed)

**. Correlation is significant at the 0.01 level (2-tailed)

Table S11. Correlation matrix for general frequency, intensity, and impairment in dimension 6. Anomalous experience of the present.

| | | frequency 6 | intensity 6 | impairment 6 |
|---|---|---|---|---|
| frequency 6 | Spearman's ρ | 1.000 | 0.898** | 0.823** |
| | p-value | | 0.000 | 0.000 |
| intensity 6 | Spearman's ρ | 0.898** | 1.000 | 0.851** |
| | p-value | 0.000 | | 0.000 |
| impairment 6 | Spearman's ρ | 0.823** | 0.851** | 1.000 |
| | p-value | 0.000 | 0.000 | |

**. Correlation is significant at the 0.01 level (2-tailed)

Published as a Supplementary Material to: A. Szuła, M. Moskalewicz, G. Stanghellini. Transdiagnostic Assessment of Temporal Experience (TATE) in Mental Disorders – Empirical Validation and Adaptation of a Structured Phenomenological Interview.

Table S12. Correlation matrix for each item's severity in dimension 6. Anomalous experience of the present.

| | | 6.a | 6.b | 6.c | 6.d | 6.e | 6.f | 6.g | 6.h | 6.i | 6.j | 6.k | 6.l | 6.m | 6.n |
|---|---|---|---|---|---|---|---|---|---|---|---|---|---|---|---|
| 6.a | Spearman's ρ | 1.000 | 0.501** | 0.301** | 0.349** | 0.423** | 0.167 | 0.478** | 0.442** | 0.670** | 0.382** | 0.175 | 0.041 | 0.226* | 0.159 |
| | p-value | | 0.000 | 0.003 | 0.000 | 0.000 | 0.100 | 0.000 | 0.000 | 0.000 | 0.000 | 0.084 | 0.686 | 0.025 | 0.118 |
| 6.b | Spearman's ρ | 0.501** | 1.000 | 0.155 | 0.398** | 0.493** | 0.166 | 0.428** | 0.447** | 0.517** | 0.304** | 0.396** | 0.361** | 0.476** | 0.322** |
| | p-value | 0.000 | | 0.128 | 0.000 | 0.000 | 0.103 | 0.000 | 0.000 | 0.000 | 0.002 | 0.000 | 0.000 | 0.000 | 0.001 |
| 6.c | Spearman's ρ | 0.301** | 0.155 | 1.000 | 0.279** | 0.132 | 0.271** | 0.270** | 0.301** | 0.326** | 0.311** | 0.254* | 0.186 | 0.110 | 0.209* |
| | p-value | 0.003 | 0.128 | | 0.005 | 0.193 | 0.007 | 0.007 | 0.003 | 0.001 | 0.002 | 0.012 | 0.066 | 0.281 | 0.039 |
| 6.d | Spearman's ρ | 0.349** | 0.398** | 0.279** | 1.000 | 0.405** | 0.403** | 0.374** | 0.275** | 0.380** | 0.569** | 0.361** | 0.430** | 0.411** | 0.266** |
| | p-value | 0.000 | 0.000 | 0.005 | | 0.000 | 0.000 | 0.000 | 0.006 | 0.000 | 0.000 | 0.000 | 0.000 | 0.000 | 0.008 |
| 6.e | Spearman's ρ | 0.423** | 0.493** | 0.132 | 0.405** | 1.000 | 0.117 | 0.333** | 0.356** | 0.295** | 0.388** | 0.251* | 0.400** | 0.381** | 0.250* |
| | p-value | 0.000 | 0.000 | 0.193 | 0.000 | | 0.250 | 0.001 | 0.000 | 0.003 | 0.000 | 0.013 | 0.000 | 0.000 | 0.013 |
| 6.f | Spearman's ρ | 0.167 | 0.166 | 0.271** | 0.403** | 0.117 | 1.000 | 0.195 | 0.244* | 0.282** | 0.472** | 0.325** | 0.316** | 0.159 | 0.115 |
| | p-value | 0.100 | 0.103 | 0.007 | 0.000 | 0.250 | | 0.055 | 0.016 | 0.005 | 0.000 | 0.001 | 0.002 | 0.118 | 0.259 |
| 6.g | Spearman's ρ | 0.478** | 0.428** | 0.270** | 0.374** | 0.333** | 0.195 | 1.000 | 0.407** | 0.472** | 0.327** | 0.318** | 0.114 | 0.381** | 0.144 |
| | p-value | 0.000 | 0.000 | 0.007 | 0.000 | 0.001 | 0.055 | | 0.000 | 0.000 | 0.001 | 0.001 | 0.263 | 0.000 | 0.159 |
| 6.h | Spearman's ρ | 0.442** | 0.447** | 0.301** | 0.275** | 0.356** | 0.244* | 0.407** | 1.000 | 0.342** | 0.303** | 0.502** | 0.154 | 0.364** | 0.422** |
| | p-value | 0.000 | 0.000 | 0.003 | 0.006 | 0.000 | 0.016 | 0.000 | | 0.001 | 0.002 | 0.000 | 0.131 | 0.000 | 0.000 |
| 6.i | Spearman's ρ | 0.670** | 0.517** | 0.326** | 0.380** | 0.295** | 0.282** | 0.472** | 0.342** | 1.000 | 0.438** | 0.306** | 0.186 | 0.270** | 0.183 |
| | p-value | 0.000 | 0.000 | 0.001 | 0.000 | 0.003 | 0.005 | 0.000 | 0.001 | | 0.000 | 0.002 | 0.067 | 0.007 | 0.071 |
| 6.j | Spearman's ρ | 0.382** | 0.304** | 0.311** | 0.569** | 0.388** | 0.472** | 0.327** | 0.303** | 0.438** | 1.000 | 0.422** | 0.390** | 0.290** | 0.246* |
| | p-value | 0.000 | 0.002 | 0.002 | 0.000 | 0.000 | 0.000 | 0.001 | 0.002 | 0.000 | | 0.000 | 0.000 | 0.004 | 0.015 |
| 6.k | Spearman's ρ | 0.175 | 0.396** | 0.254* | 0.361** | 0.251* | 0.325** | 0.318** | 0.502** | 0.306** | 0.422** | 1.000 | 0.349** | 0.425** | 0.336** |
| | p-value | 0.084 | 0.000 | 0.012 | 0.000 | 0.013 | 0.001 | 0.001 | 0.000 | 0.002 | 0.000 | | 0.000 | 0.000 | 0.001 |
| 6.l | Spearman's ρ | 0.041 | 0.361** | 0.186 | 0.430** | 0.400** | 0.316** | 0.114 | 0.154 | 0.186 | 0.390** | 0.349** | 1.000 | 0.477** | 0.341** |
| | p-value | 0.686 | 0.000 | 0.066 | 0.000 | 0.000 | 0.002 | 0.263 | 0.131 | 0.067 | 0.000 | 0.000 | | 0.000 | 0.001 |
| 6.m | Spearman's ρ | 0.226* | 0.476** | 0.110 | 0.411** | 0.381** | 0.159 | 0.381** | 0.364** | 0.270** | 0.290** | 0.425** | 0.477** | 1.000 | 0.308** |
| | p-value | 0.025 | 0.000 | 0.281 | 0.000 | 0.000 | 0.118 | 0.000 | 0.000 | 0.007 | 0.004 | 0.000 | 0.000 | | 0.002 |
| 6.n | Spearman's ρ | 0.159 | 0.322** | 0.209* | 0.266** | 0.250* | 0.115 | 0.144 | 0.422** | 0.183 | 0.246* | 0.336** | 0.341** | 0.308** | 1.000 |
| | p-value | 0.118 | 0.001 | .039 | 0.008 | 0.013 | 0.259 | 0.159 | 0.000 | 0.071 | 0.015 | 0.001 | 0.001 | 0.002 | |

*. Correlation is significant at the 0.05 level (2-tailed)

**. Correlation is significant at the 0.01 level (2-tailed)

Table S13. Correlation matrix for general frequency, intensity, and impairment in dimension 7. Anomalous experience of the future.

| | | frequency 7 | intensity 7 | impairment 7 |
|---|---|---|---|---|
| frequency 7 | Spearman's ρ | 1.000 | 0.917** | 0.820** |
| | p-value | | 0.000 | 0.000 |
| intensity 7 | Spearman's ρ | 0.917** | 1.000 | 0.869** |
| | p-value | 0.000 | | 0.000 |
| impairment 7 | Spearman's ρ | 0.820** | 0.869** | 1.000 |
| | p-value | 0.000 | 0.000 | |

**. Correlation is significant at the 0.01 level (2-tailed)

Table S14. Correlation matrix for each item's severity in dimension 7. Anomalous experience of the future.

| | | 7.a | 7.b | 7.c | 7.d | 7.e | 7.f | 7.g |
|---|---|---|---|---|---|---|---|---|
| 7.a | Spearman's ρ | 1.000 | 0.130 | 0.355** | 0.024 | 0.354** | 0.320** | 0.255* |
| | p-value | | 0.200 | 0.000 | 0.813 | 0.000 | 0.001 | 0.011 |
| 7.b | Spearman's ρ | 0.130 | 1.000 | 0.219* | 0.550** | 0.156 | 0.258* | 0.159 |
| | p-value | 0.200 | | 0.031 | 0.000 | 0.126 | 0.010 | 0.118 |
| 7.c | Spearman's ρ | 0.355** | 0.219* | 1.000 | 0.315** | 0.160 | 0.232* | 0.250* |
| | p-value | 0.000 | 0.031 | | 0.002 | 0.115 | 0.021 | 0.013 |
| 7.d | Spearman's ρ | 0.024 | 0.550** | 0.315** | 1.000 | 0.066 | 0.472** | 0.327** |
| | p-value | 0.813 | 0.000 | 0.002 | | 0.518 | 0.000 | 0.001 |
| 7.e | Spearman's ρ | 0.354** | 0.156 | 0.160 | 0.066 | 1.000 | 0.390** | 0.354** |
| | p-value | 0.000 | 0.126 | 0.115 | 0.518 | | 0.000 | 0.000 |
| 7.f | Spearman's ρ | 0.320** | 0.258* | 0.232* | 0.472** | 0.390** | 1.000 | 0.511** |
| | p-value | 0.001 | 0.010 | 0.021 | 0.000 | 0.000 | | 0.000 |
| 7.g | Spearman's ρ | 0.255* | 0.159 | 0.250* | 0.327** | 0.354** | 0.511** | 1.000 |
| | p-value | 0.011 | 0.118 | 0.013 | 0.001 | 0.000 | 0.000 | |

*. Correlation is significant at the 0.05 level (2-tailed)
**. Correlation is significant at the 0.01 level (2-tailed)

Table S15. Correlation matrix for frequency, intensity, and impairment in all dimensions.

| | | general frequency | general intensity | general impairment |
|---|---|---|---|---|
| general frequency | Spearman's ρ | 1.000 | 0.941** | 0.902** |
| | p-value | | 0.000 | 0.000 |
| general intensity | Spearman's ρ | 0.941** | 1.000 | 0.927** |
| | p-value | 0.000 | | 0.000 |
| general impairment | Spearman's ρ | 0.902** | 0.927** | 1.000 |
| | p-value | .000 | 0.000 | |

**. Correlation is significant at the 0.01 level (2-tailed)
